# Supplementary material for: A Combined GLP-1/PPARa/CB1-Based Therapy to Restore the Central and Peripheral Metabolic Dysregulation Induced by a High-Fructose High-Fat Diet
Source: Int J Mol Sci. 2025 Mar 7;26(6):2420. doi: 10.3390/ijms26062420 (PMC11942104; doi:10.3390/ijms26062420)
Supplement: Supplementary file 1 [file ijms-26-02420-s001.zip › ijms-3479564-supplementary.pdf]

Supplementary materials for:

Combined OLHHA and Liraglutide treatment effectively moderates peripheral and central dysregulations in a preclinical model of obesity induced by high-fructose high-fat diet

Marialuisa de Ceglia[1,2*], Nabila Rasheed[3], Rubén Tovar[1], Inés Pareja Cerbán[1], Andrea Arias Sáez[1], Ana Gavito[1], Silvana Gaetani[3], Carlo Cifani[2], Fernando Rodríguez de Fonseca[1], Juan Decara[1*]

Table 1. Parameters of two-way ANOVA for peripheral measurements

Parameter	Factor	F	p	Degrees of freedom
Body weight gain	diet	132,202	***p<0.001***	39
	treatm	10,011	***p<0.001***	
Triglycerides	diet	9,984	***p<0.01***	50
	treatm	21,128	***p<0.001***	
Cholesterol	diet	0,889	0,350	62
	treatm	0,320	0,811	
HDL	diet	0,141	0,710	44
	treatm	1,950	0,137	
LDL	diet	8,965	***p<0.01***	36
	treatm	9,174	***p<0.001***	
AST	diet	1,245	0,273	37
	treatm	8,063	***p<0.001***	
ALT	diet	3,719	0,063	35
	treatm	6,169	***p<0.01***	
Bilirubin	diet	10,086	***p<0.01***	44
	treatm	4,452	***p<0.01***	

Table 2. Parameters of two-way ANOVA for protein expression in HYPO

Protein expression	Factor	F	p	Degrees of freedom
CB1	diet	7,631	***p<0,05***	38
	treatm	1,600	,208	
CB2	diet	,601	,444	37
	treatm	10,223	***p<0,001***	
PPARα	diet	2,653	,115	32
	treatm	15,668	***p<0,001***	
DAGLα	diet	5,713	***p<0,05***	35
	treatm	0,578	,634	
DAGLβ	diet	9,759	***p<0,01***	34
	treatm	10,159	***p<0,001***	
MAGL	diet	10,786	***p<0,01***	38
	treatm	4,878	***p<0,05***	
NAPE-PLD	diet	22,251	***p<0,001***	37
	treatm	3,858	***p<0,01***	
FAAH	diet	4,422	***p<0,05***	34

	treatm	3,491	***p<0,05***	
GLP1-R	diet	4,393	***p<0,05***	38
	treatm	9,905	***p<0,001***	
MOR	diet	15,087	,351	36
	treatm	1,132	***p<0,01***	
NPY1-R	diet	12,777	***p<0,01***	37
	treatm	18,330	***p<0,001***	
IRβ	diet	3,668	,064	38
	treatm	9,996	***p<0,001***	
IRS-1	diet	,433	,515	38
	treatm	4,314	***p<0,05***	
IRS-1 phospho TYR	diet	,400	,531	38
	treatm	5,829	***p<0,01***	
IRS-1 phospho SER	diet	,284	,598	39
	treatm	2,375	,087	
PI3K	diet	13,459	***p<0,01***	39
	treatm	2,047	,125	
phospho PI3K	diet	4,755	***p<0,05***	39
	treatm	2,084	,120	
AKT	diet	,560	,460	37
	treatm	4,213	***p<0,05***	
phospho AKT	diet	,967	,332	39
	treatm	0,694	,562	
GSK3β	diet	48,504	***p<0,001***	39
	treatm	0,544	,655	
phospho GSK3β	diet	6,688	***p<0,05***	39
	treatm	0,122	,947	
mTOR	diet	8,837	***p<0,01***	39
	treatm	5,234	***p<0,01***	
phospho mTOR	diet	1,734	*,196*	39
	treatm	2,806	***p<0,05***	
ERK1	diet	1,581	,217	38
	treatm	2,445	,081	
phospho ERK1	diet	5,903	***p<0,05***	33
	treatm	0,636	,598	
ERK2	diet	13,391	***p<0,01***	37
	treatm	6,507	***p<0,01***	
phospho ERK2	diet	14,891	***p<0,01***	36
	treatm	6,656	***p<0,01***	
TAU	diet	1,043	,315	35
	treatm	5,078	***p<0,05***	
AT8	diet	1,617	,213	36
	treatm	0,485	,695	
AT100	diet	,929	,343	34
	treatm	0,818	,494	
CDK5	diet	46,557	***p<0,001***	39
	treatm	0,089	,965	
P35	diet	56,532	***p<0,001***	37
	treatm	7,627	***p<0,01***	
P25	diet	11,147	***p<0,01***	39
	treatm	11,852	***p<0,001***	
BDNF	diet	2,276	,141	36
	treatm	6,268	***p<0,01***	
TrkB	diet	8,284	***p<0,05***	38
	treatm	10,965	***p<0,001***	
GFAP	diet	,353	,556	39
	treatm	6,007	***p<0,01***	
IBA-1	diet	3,824	***p<0,05***	37
	treatm	4,798	***p<0,05***	
phospho NF-kB	diet	3,793	***p<0,05***	36
	treatm	8,515	***p<0,001***	
NF-kB	diet	5,086	***p<0,05***	39

	treatm	10,183	***p<0,001***	
TNFα	diet	4,625	***p<0,05***	38
	treatm	1,218	,318	
FosB	diet	18,446	***p<0,001***	38
	treatm	3,756	***p<0,05***	
ΔFosB	diet	4,837	***p<0,05***	37
	treatm	4,451	***p<0,05***	

Table 3. Parameters of two-way ANOVA for protein expression in HIPPO

Protein expression	Factor	F	p	Degrees of freedom
CB1	diet	,068	,796	39
	treatm	3,967	***p<0,05***	
CB2	diet	2,816	,103	36
	treatm	0,145	,932	
PPARα	diet	,013	,910	39
	treatm	0,740	,536	
DAGLα	diet	7,159	***p<0,05***	35
	treatm	1,013	,400	
DAGLβ	diet	4,806	***p<0,05***	35
	treatm	3,151	***p<0,05***	
MAGL	diet	15,427	***p<0,001***	39
	treatm	6,250	***p<0,01***	
NAPE-PLD	diet	,041	,841	38
	treatm	8,346	***p<0,001***	
FAAH	diet	6,043	***p<0,05***	39
	treatm	2,398	***p<0,05***	
GLP1-R	diet	1,302	,262	38
	treatm	7,559	***p<0,01***	
MOR	diet	,133	,718	36
	treatm	4,404	***p<0,05***	
NPY1-R	diet	2,509	,122	38
	treatm	2,156	,111	
IRβ	diet	33,788	***p<0,001***	39
	treatm	0,385	,764	
IRS-1	diet	11,201	***p<0,01***	38
	treatm	4,052	***p<0,05***	
IRS-1 phospho TYR	diet	26,241	***p<0,001***	35
	treatm	3,806	***p<0,05***	
IRS-1 phospho SER	diet	8,754	***p<0,01***	34
	treatm	0,164	,920	
PI3K	diet	1,079	,308	31
	treatm	0,667	,579	
phospho PI3K	diet	5,514	***p<0,05***	29
	treatm	0,924	,444	
AKT	diet	,842	,365	39
	treatm	0,734	,539	
phospho AKT	diet	,174	,680	29
	treatm	0,483	,697	
GSK3β	diet	,099	,755	31
	treatm	0,570	,639	
phospho GSK3β	diet	1,147	,294	31
	treatm	0,281	,839	
mTOR	diet	1,213	,280	30
	treatm	2,670	***p<0,05***	
phospho mTOR	diet	9,527	***p<0,01***	30
	treatm	0,330	,803	
ERK1	diet	1,664	,209	28
	treatm	1,735	,187	
phospho ERK1	diet	,110	,743	27

	treatm	9,835	***p<0,001***	
ERK2	diet	9,625	***p<0,01***	28
	treatm	0,165	,919	
phospho ERK2	diet	8,659	***p<0,01***	28
	treatm	3,882	***p<0,05***	
AT8	diet	,883	,354	37
	treatm	3,073	***p<0,05***	
AT100	diet	,010	,921	38
	treatm	2,167	,110	
TAU	diet	4,578	***p<0,05***	39
	treatm	3,096	***p<0,05***	
CDK5	diet	17,367	***p<0,001***	39
	treatm	3,259	***p<0,05***	
p35	diet	50,769	***p<0,001***	37
	treatm	4,479	***p<0,05***	
25	diet	2,427	,129	36
	treatm	6,115	***p<0,01***	
BDNF	diet	,056	,815	39
	treatm	1,016	,397	
TrkB	diet	10,727	***p<0,01***	39
	treatm	,195	,899	
GFAP	diet	2,928	***p<0,05***	30
	treatm	8,540	***p<0,001***	
IBA-1	diet	,590	,449	31
	treatm	1,095	,368	
phospho NF-kB	diet	8,938	***p<0,01***	37
	treatm	2,962	***p<0,05***	
NF-kB	diet	2,111	,155	38
	treatm	2,015	,130	
TNFα	diet	6,149	***p<0,05***	35
	treatm	3,582	***p<0,05***	
FosB	diet	3,211	***p<0,05***	38
	treatm	0,406	,750	
ΔFosB	diet	5,083	***p<0,05***	39
	treatm	0,190	,902	

Table 4. Parameters of two-way ANOVA for protein expression in PFC

Protein expression	Factor	F	p	Degrees of freedom
CB1	diet	2,118	,155	38
	treatm	1,133	,350	
CB2	diet	51,192	***p<0,001***	39
	treatm	2,951	***p<0,05***	
PPARα	diet	40,613	***p<0,001***	36
	treatm	14,013	***p<0,001***	
DAGLα	diet	17,902	***p<0,001***	38
	treatm	4,289	***p<0,05***	
DAGLβ	diet	9,127	***p<0,01***	38
	treatm	5,064	***p<0,01***	
MAGL	diet	57,103	***p<0,001***	39
	treatm	8,118	***p<0,001***	
NAPE-PLD	diet	15,681	***p<0,001***	38
	treatm	1,510	,230	
FAAH	diet	4,272	***p<0,05***	39
	treatm	6,005	***p<0,01***	
GLP1-R	diet	,925	,343	38
	treatm	6,447	***p<0,01***	
MOR	diet	44,784	***p<0,001***	37
	treatm	4,869	***p<0,01***	
NPY1-R	diet	,247	,622	38
	treatm	2,332	***p<0,05***	

IRβ	diet	,003	,954	37
	treatm	2,559	***p<0,05***	
IRS-1	diet	132,091	***p<0,001***	31
	treatm	7,209	***p<0,01***	
IRS-1 phospho TIR	diet	1,166	,290	30
	treatm	2,196	,113	
IRS-1 phospho SER	diet	15,273	***p<0,01***	29
	treatm	15,628	***p<0,001***	
PI3K	diet	2,908	,100	31
	treatm	0,440	,726	
phospho PI3K	diet	24,606	***p<0,001***	31
	treatm	0,232	,873	
AKT	diet	,871	,357	39
	treatm	2,240	,101	
phospho AKT	diet	9,653	***p<0,01***	30
	treatm	6,119	***p<0,01***	
GSK3β	diet	2,991	***p<0,05***	31
	treatm	5,283	***p<0,01***	
phospho GSK3β	diet	1,409	,247	28
	treatm	1,315	,293	
mTOR	diet	65,214	***p<0,001***	31
	treatm	3,324	***p,0,05***	
phospho mTOR	diet	1,054	,312	39
	treatm	0,679	,571	
ERK1	diet	33,477	***p<0,001***	31
	treatm	42,487	***p<0,001***	
phospho ERK1	diet	6,389	***p<0,05***	29
	treatm	2,534	***p<0,05***	
ERK2	diet	35,797	***p<0,001***	31
	treatm	1,459	,248	
phospho ERK2	diet	,512	,481	29
	treatm	0,623	,607	
AT8	diet	10,318	***p<0,01***	36
	treatm	2,950	***p<0,05***	
AT100	diet	,039	,845	35
	treatm	1,809	,166	
TAU	diet	26,854	***p<0,001***	37
	treatm	1,963	,139	
CDK5	diet	8,034	***p<0,01***	38
	treatm	3,546	***p<0,05***	
p35	diet	2,043	,162	39
	treatm	4,514	***p<0,01***	
p25	diet	25,648	***p<0,001***	39
	treatm	0,210	,888	
BDNF	diet	1,729	,197	38
	treatm	1,155	,341	
TrkB	diet	,038	,847	38
	treatm	0,804	,500	
GFAP	diet	5,214	***p<0,05***	30
	treatm	1,015	,402	
IBA-1	diet	3,245	***p<0,05***	31
	treatm	2,548	***p<0,05***	
phospho NF-kB	diet	,529	,472	37
	treatm	0,489	,692	
NF-kB	diet	2,629	,114	37
	treatm	1,379	,266	
TNFα	diet	11,430	***p<0,01***	23
	treatm	0,547	,656	
ΔFosB	diet	1,494	,230	38
	treatm	0,852	,475	
FosB	diet	7,237	***p<0,05***	36
	treatm	0,873	,465	

Supplementary figure S1: Western Blots of HYPO, Ponceau and staining of membranes.

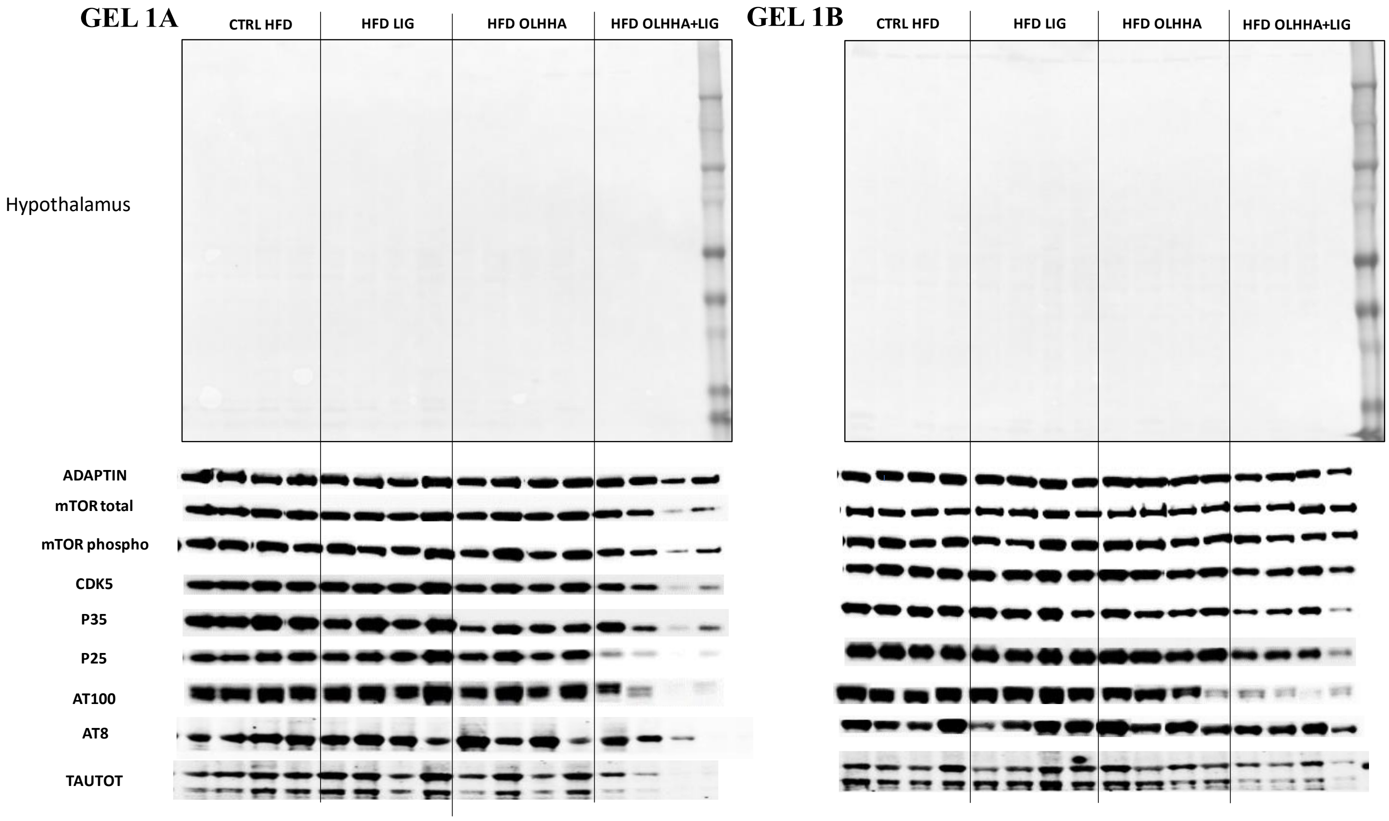

Supplementary figure S2: Western Blots of HYPO, Ponceau and staining of membranes.

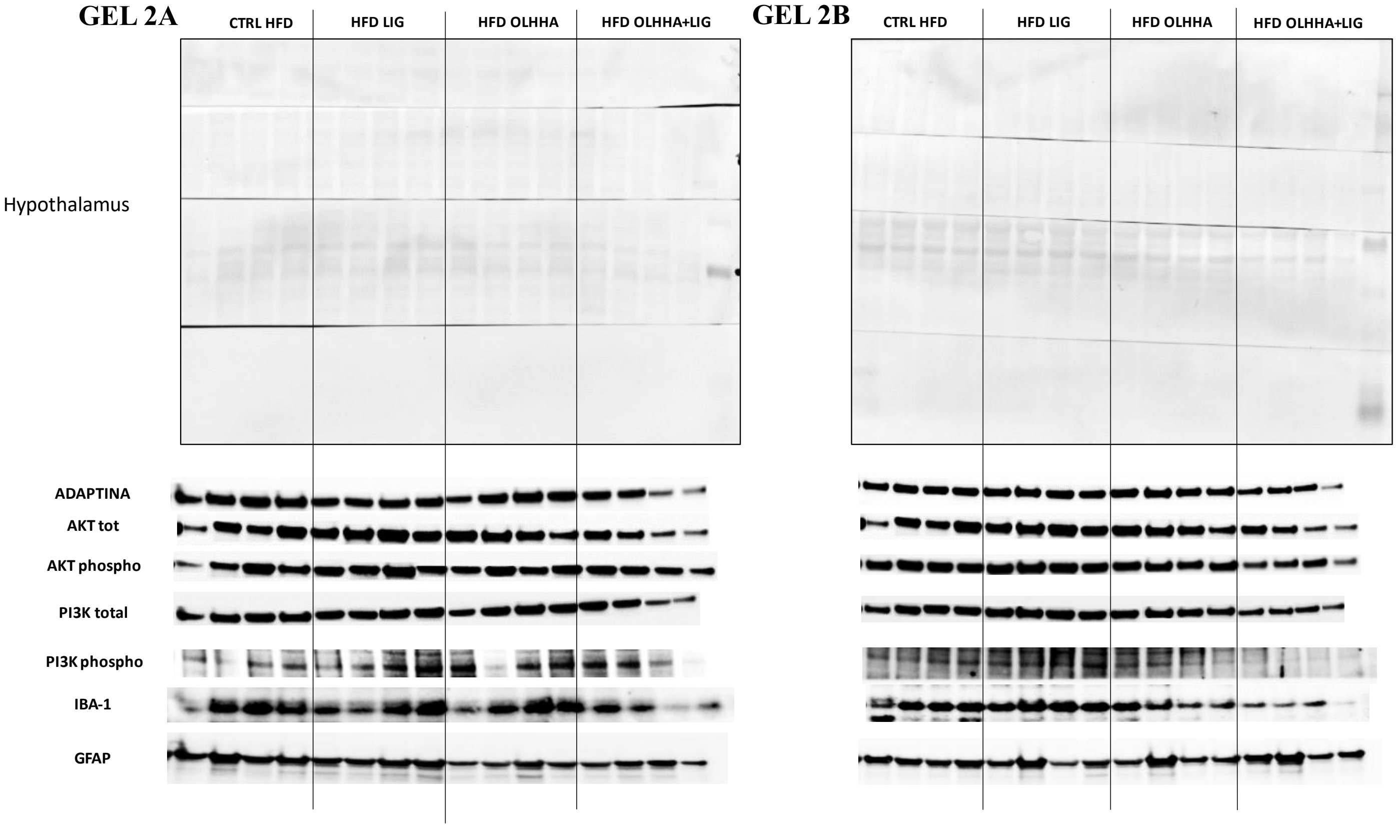

Supplementary figure S3: Western Blots of HYPO, Ponceau and staining of membranes.

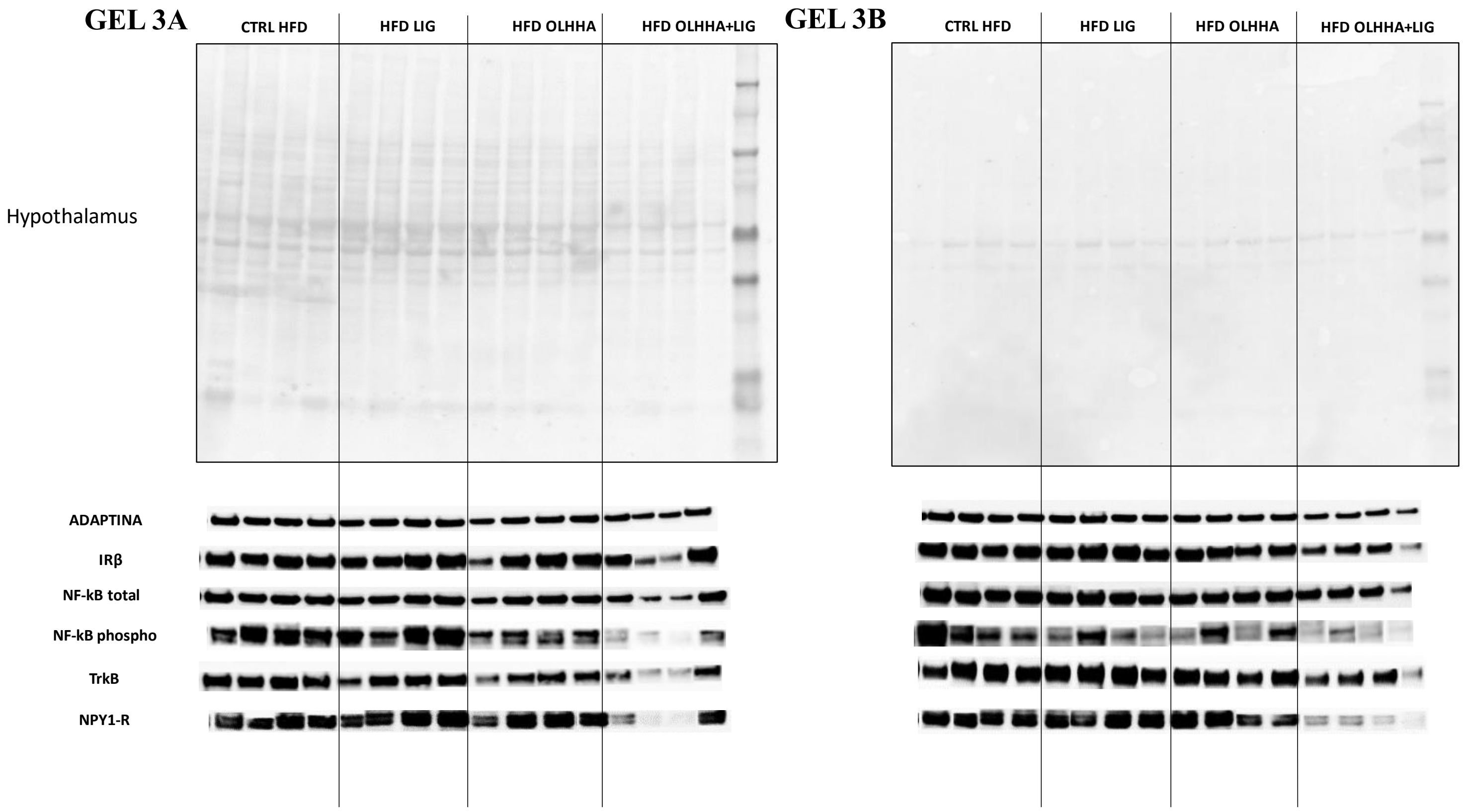

Supplementary figure S4: Western Blots of HYPO, Ponceau and staining of membranes.

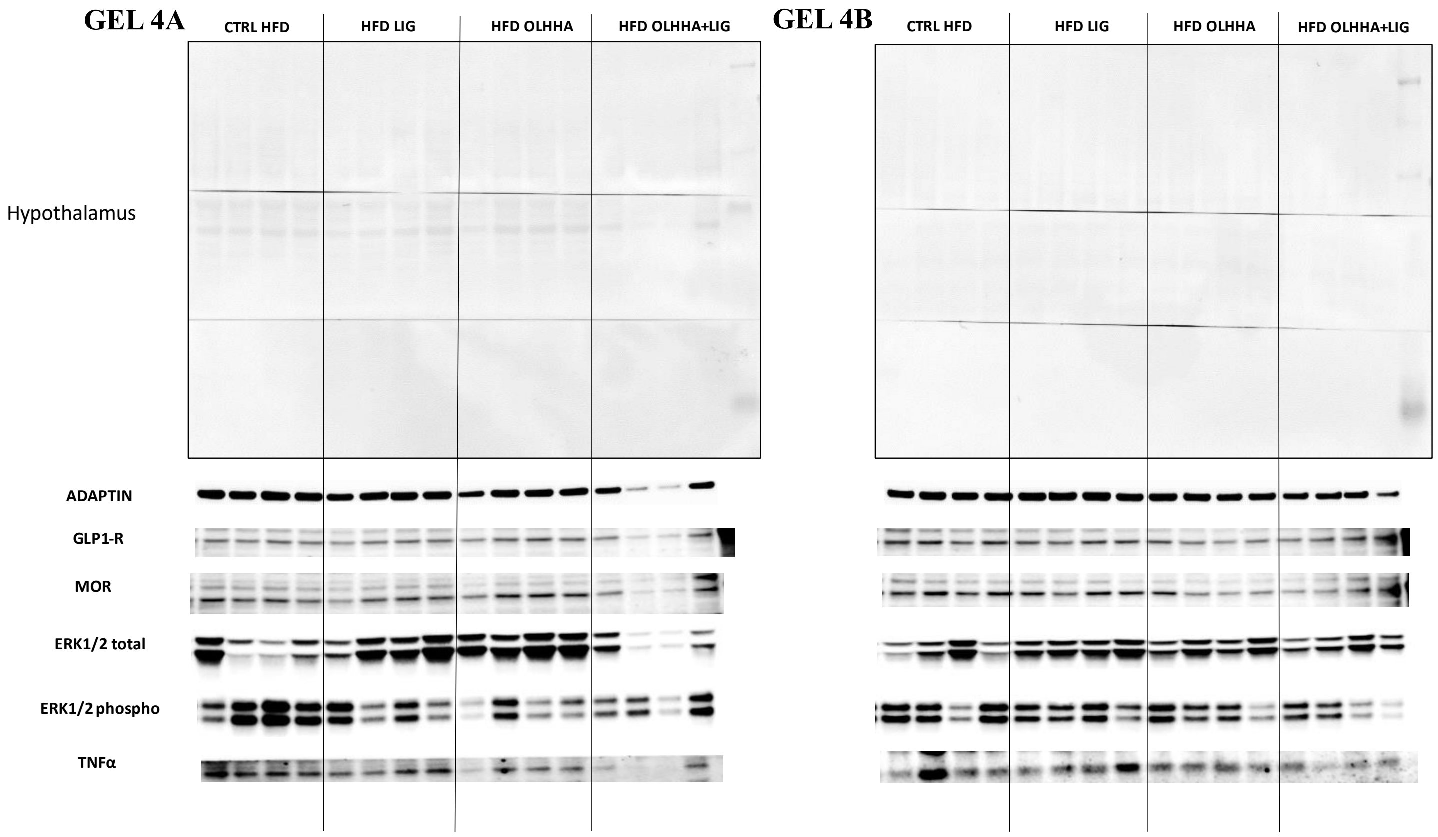

Supplementary figure S5: Western Blots of HYPO, Ponceau and staining of membranes.

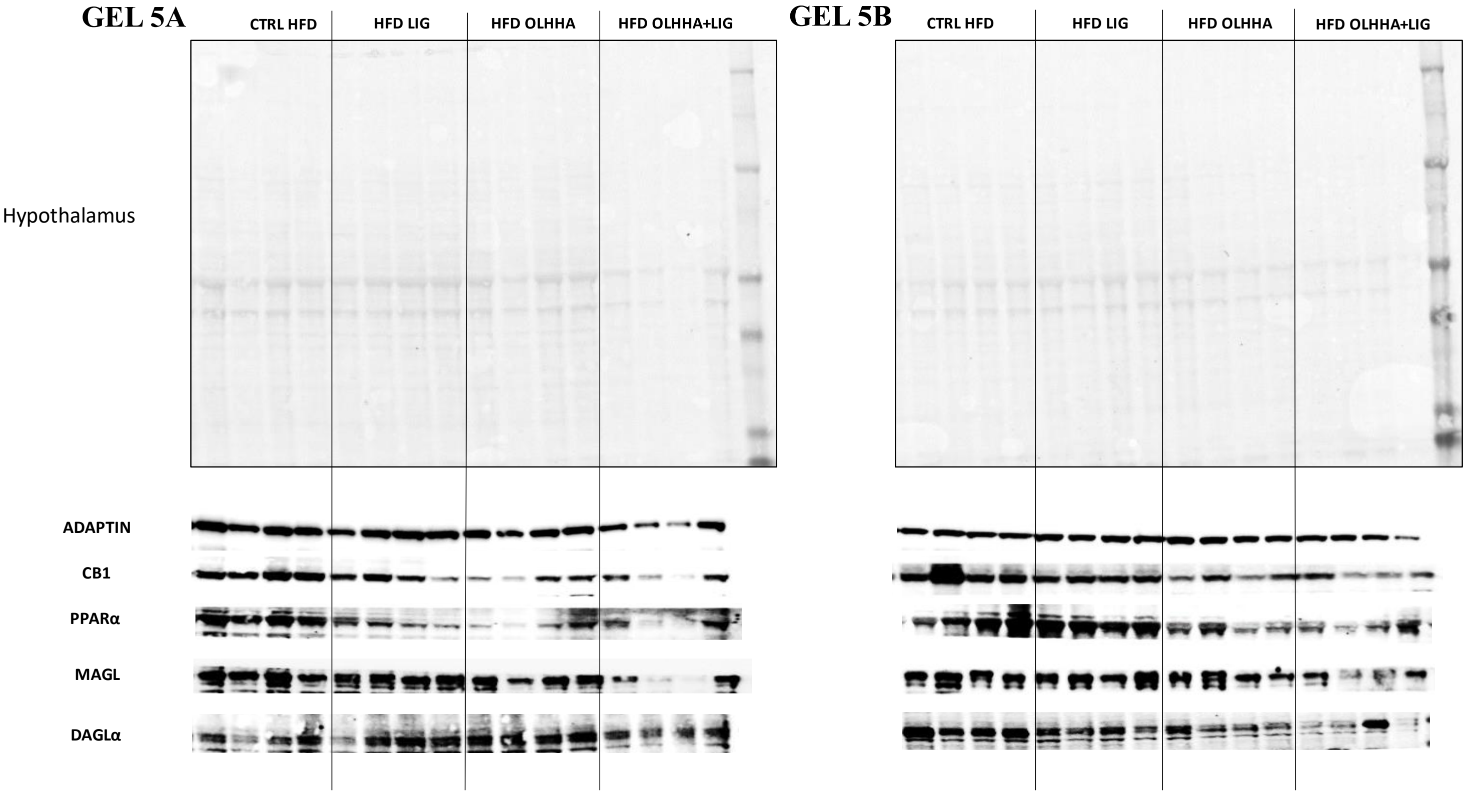

Supplementary figure S6: Western Blots of HYPO, Ponceau and staining of membranes.

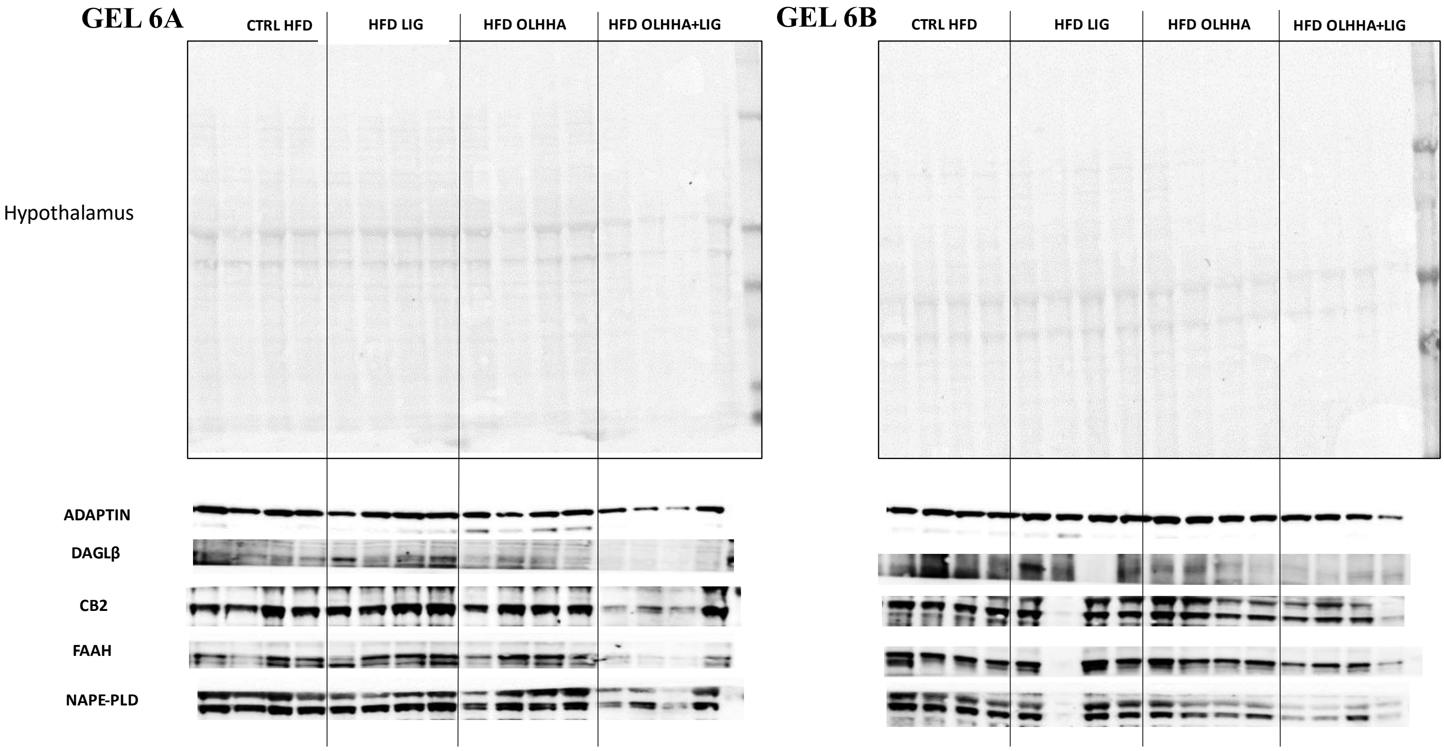

Supplementary figure S7: Western Blots of HYPO, Ponceau and staining of membranes.

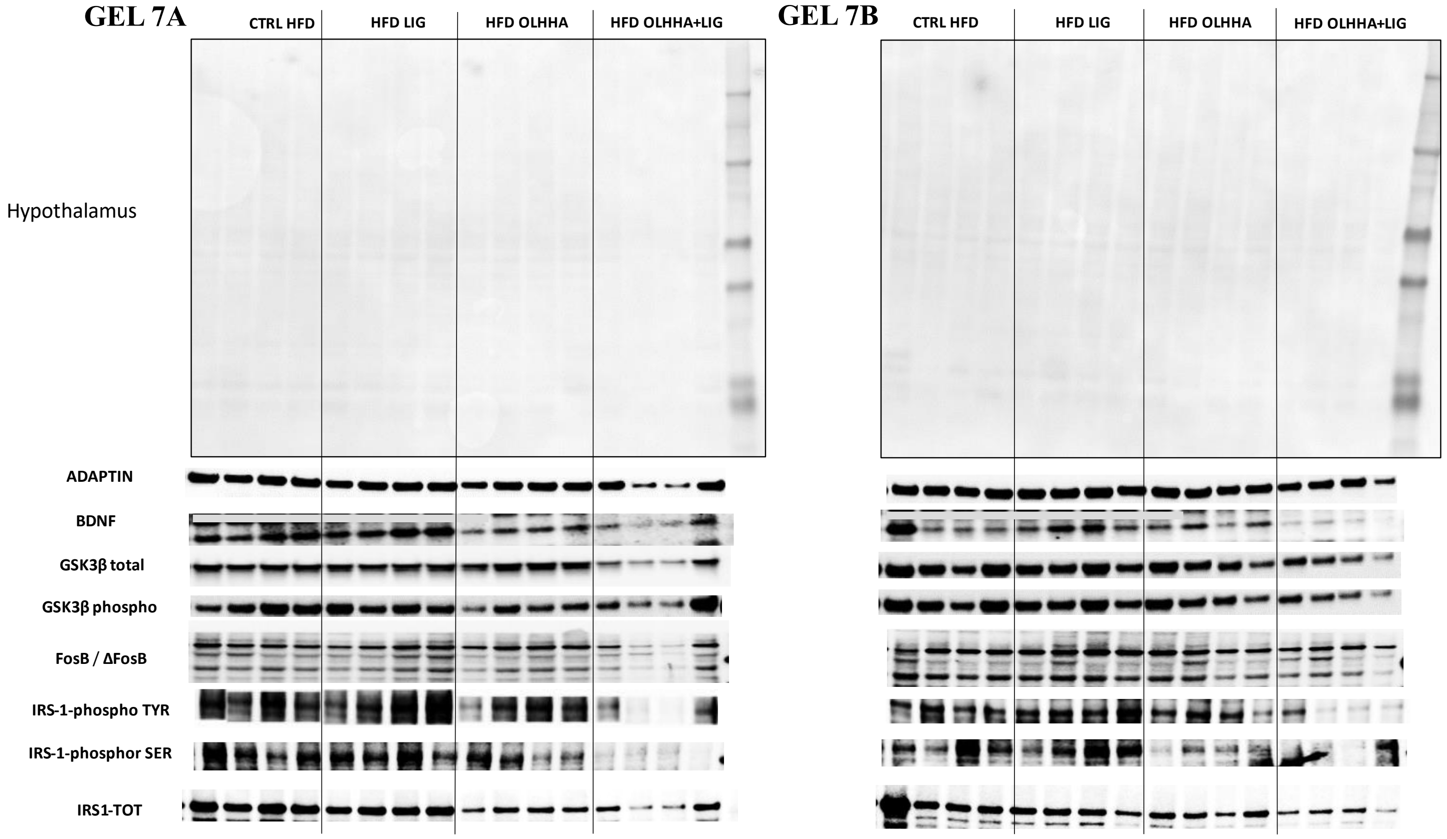

Supplementary figure S8: Western Blots of HIPPO, Ponceau and staining of membranes.

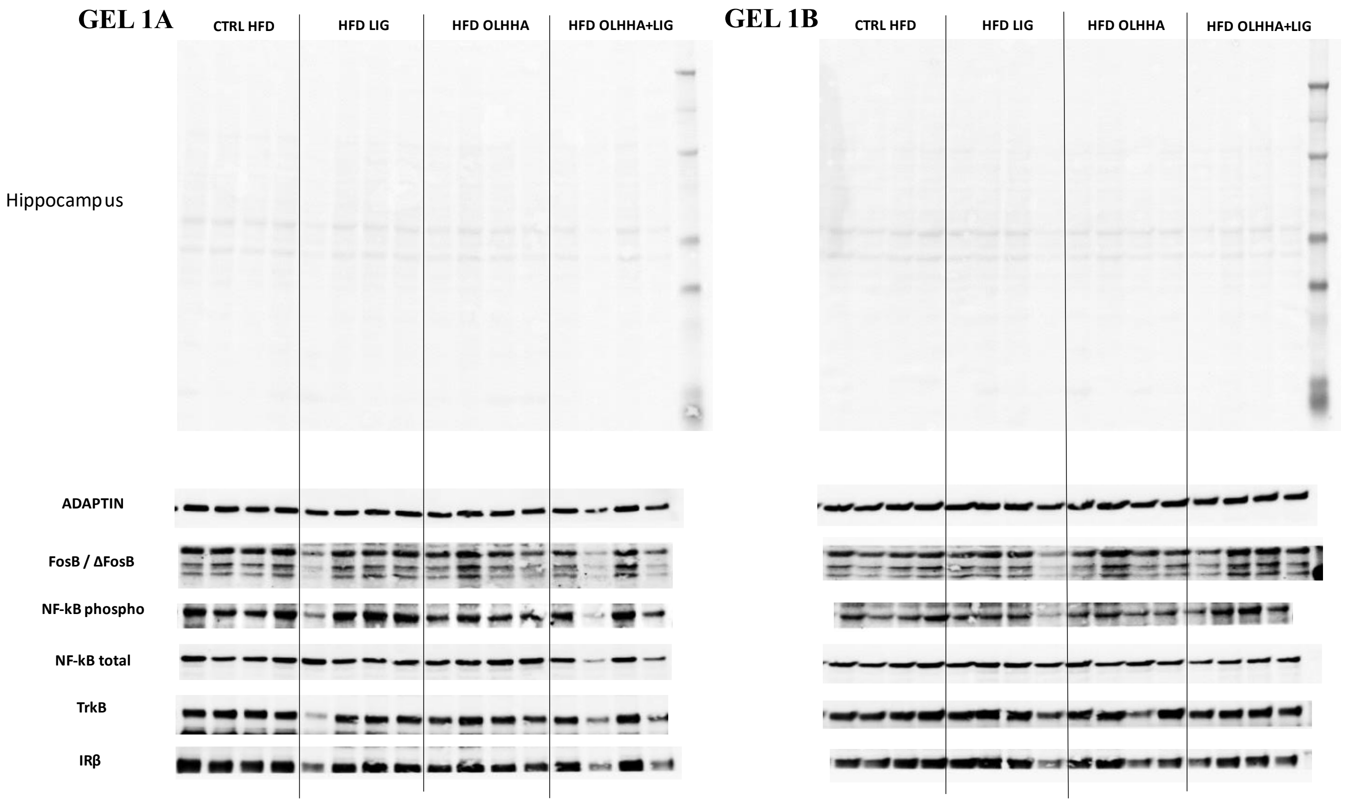

Supplementary figure S9: Western Blots of HIPPO, Ponceau and staining of membranes.

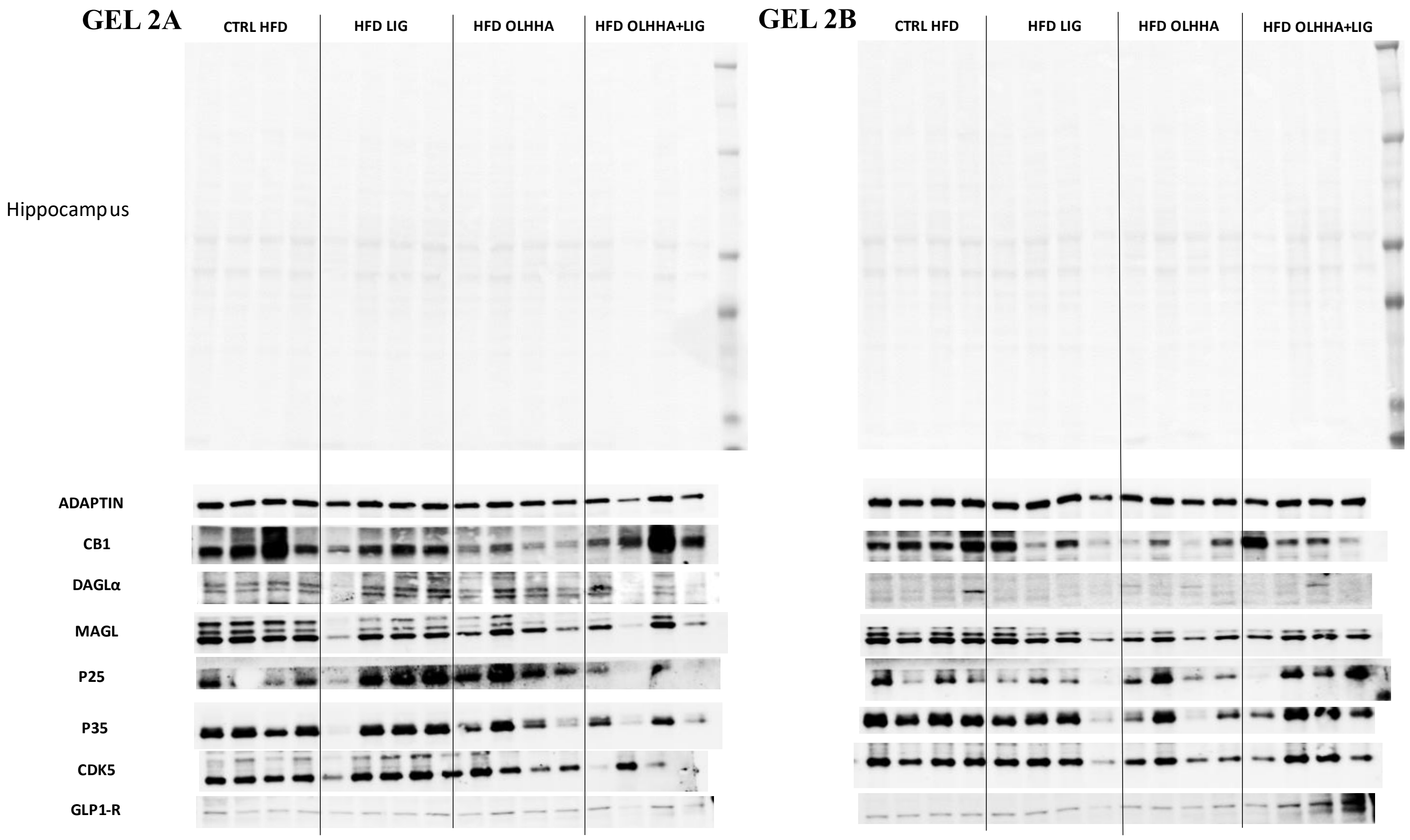

Supplementary figure S10: Western Blots of HIPPO, Ponceau and staining of membranes.

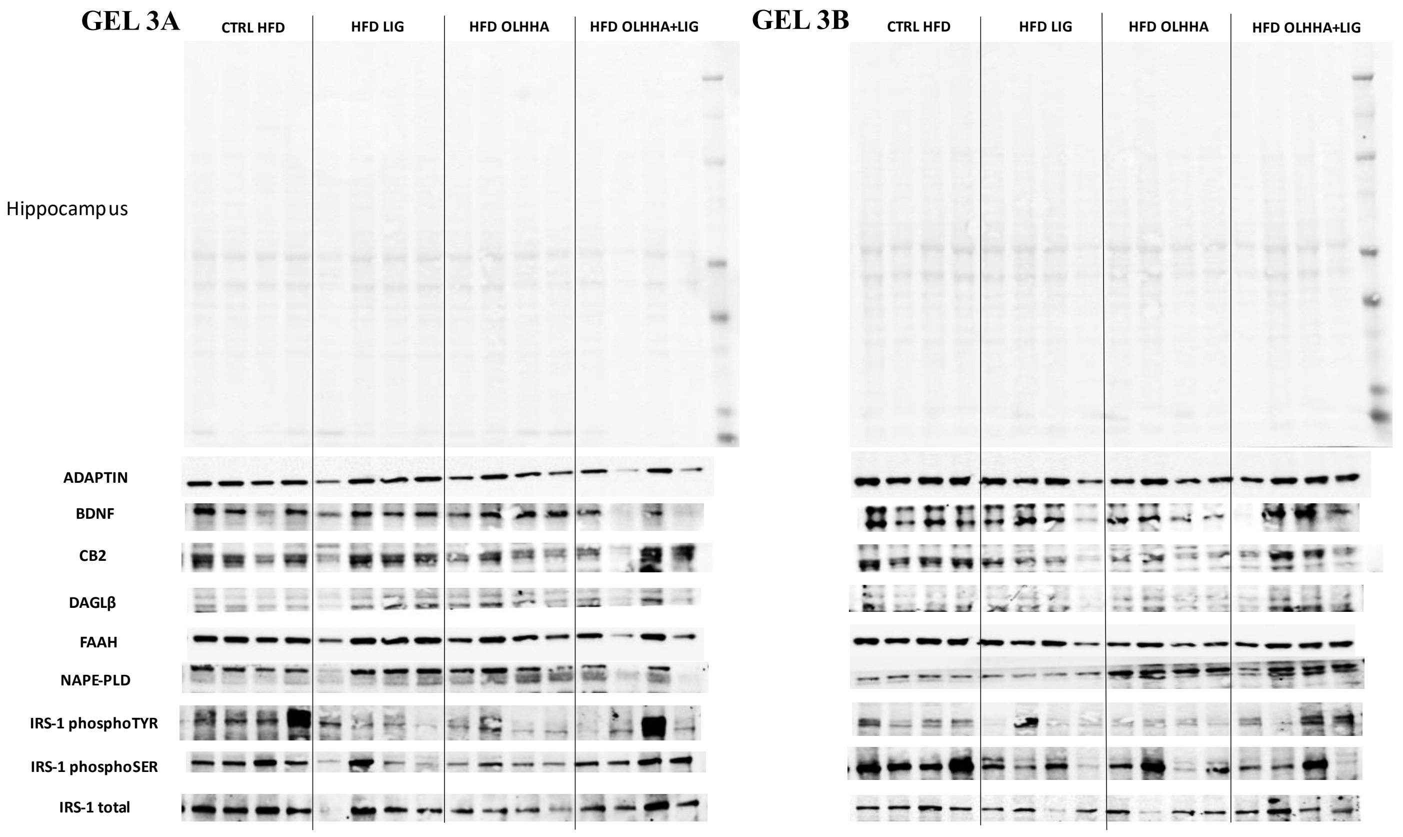

Supplementary figure S11: Western Blots of HIPPO, Ponceau and staining of membranes.

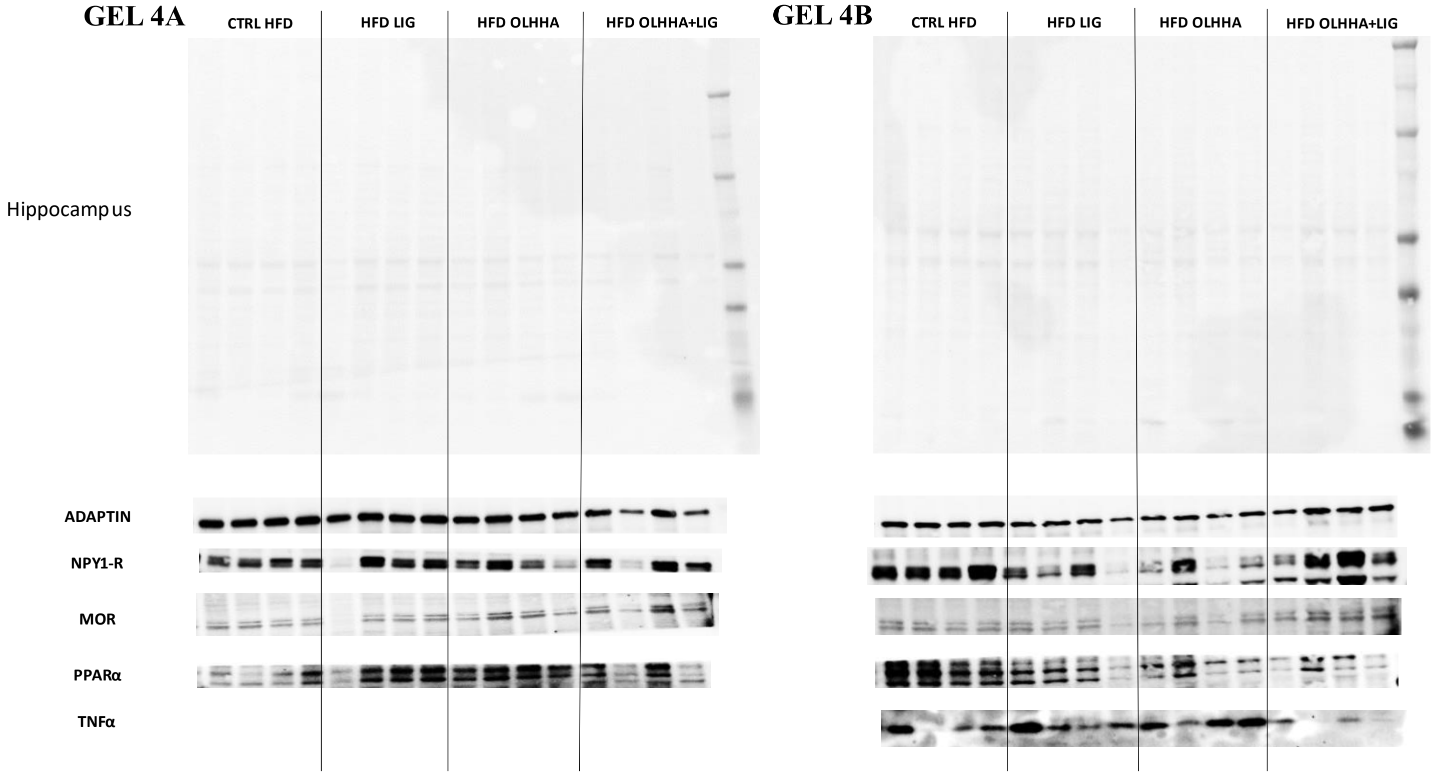

Supplementary figure S12: Western Blots of HIPPO, Ponceau and staining of membranes.

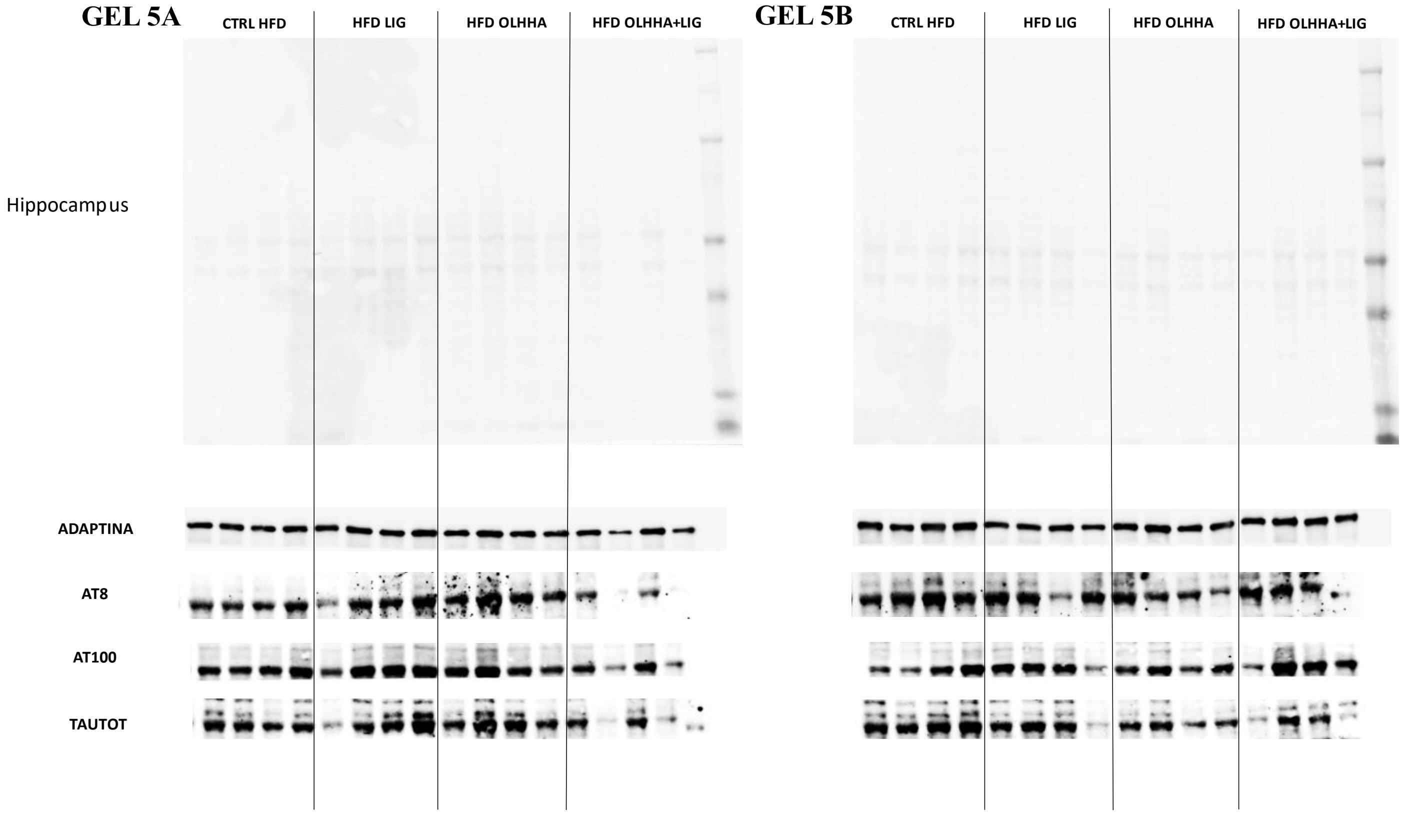

Supplementary figure S13: Western Blots of HIPPO, Ponceau and staining of membranes.

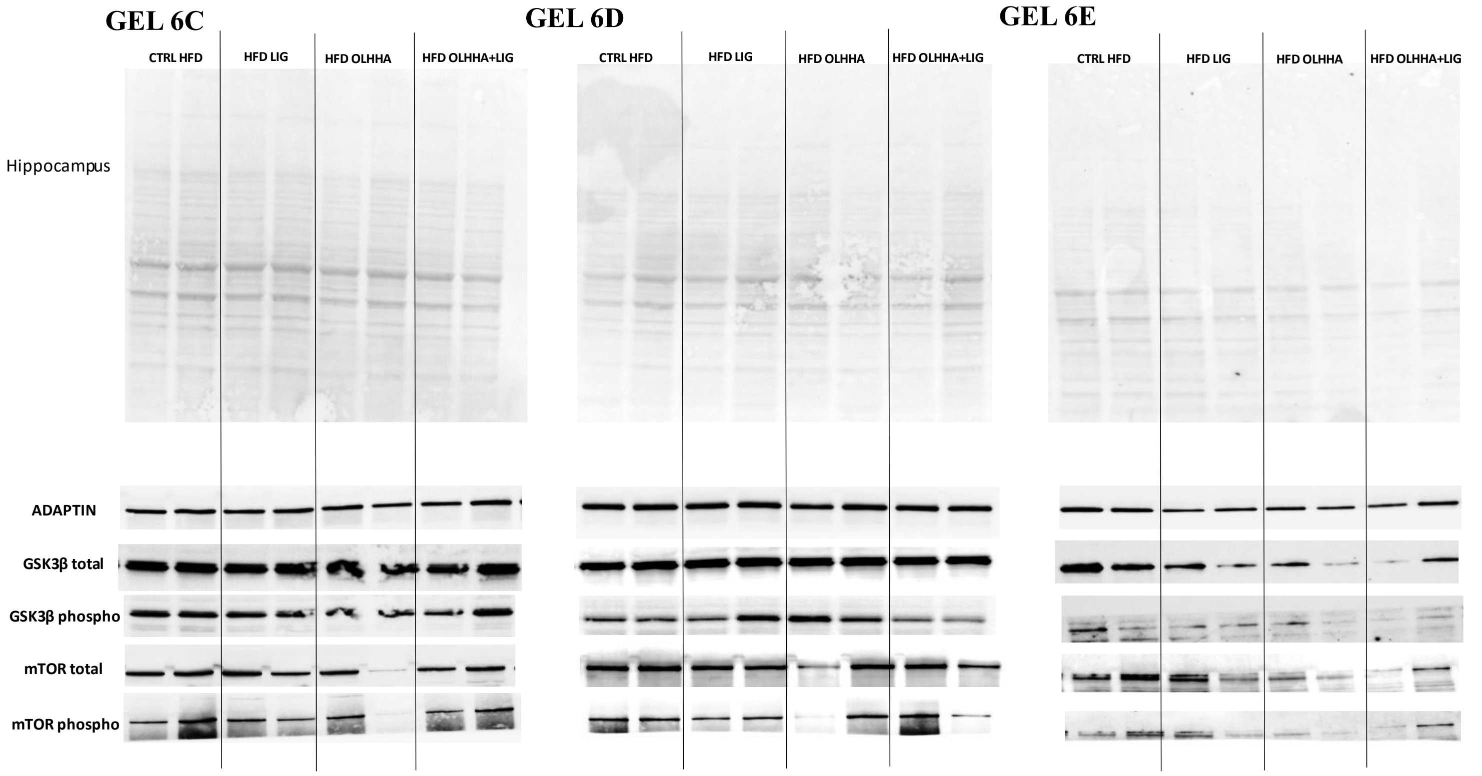

Supplementary figure S14: Western Blots of HIPPO, Ponceau and staining of membranes.

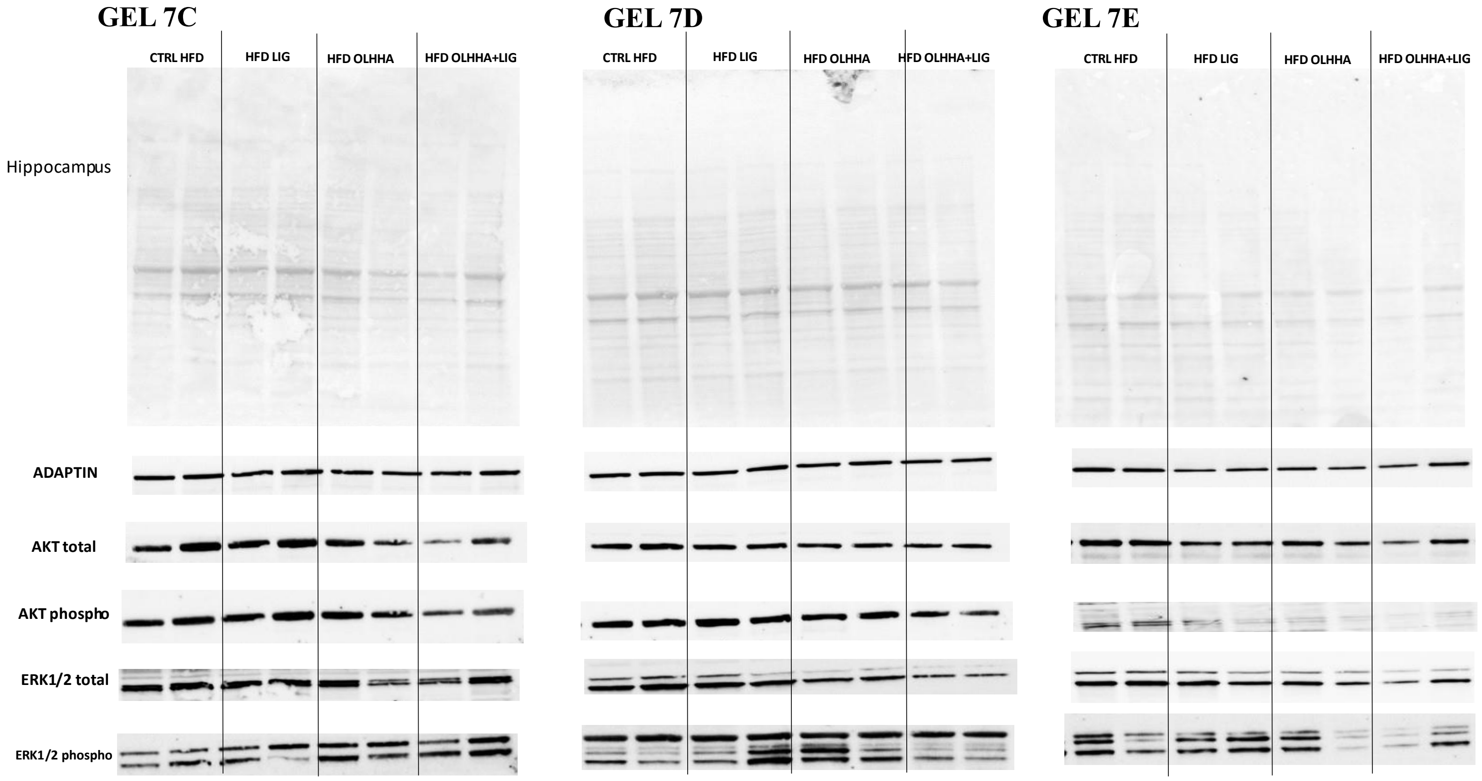

Supplementary figure S15: Western Blots of HIPPO, Ponceau and staining of membranes.

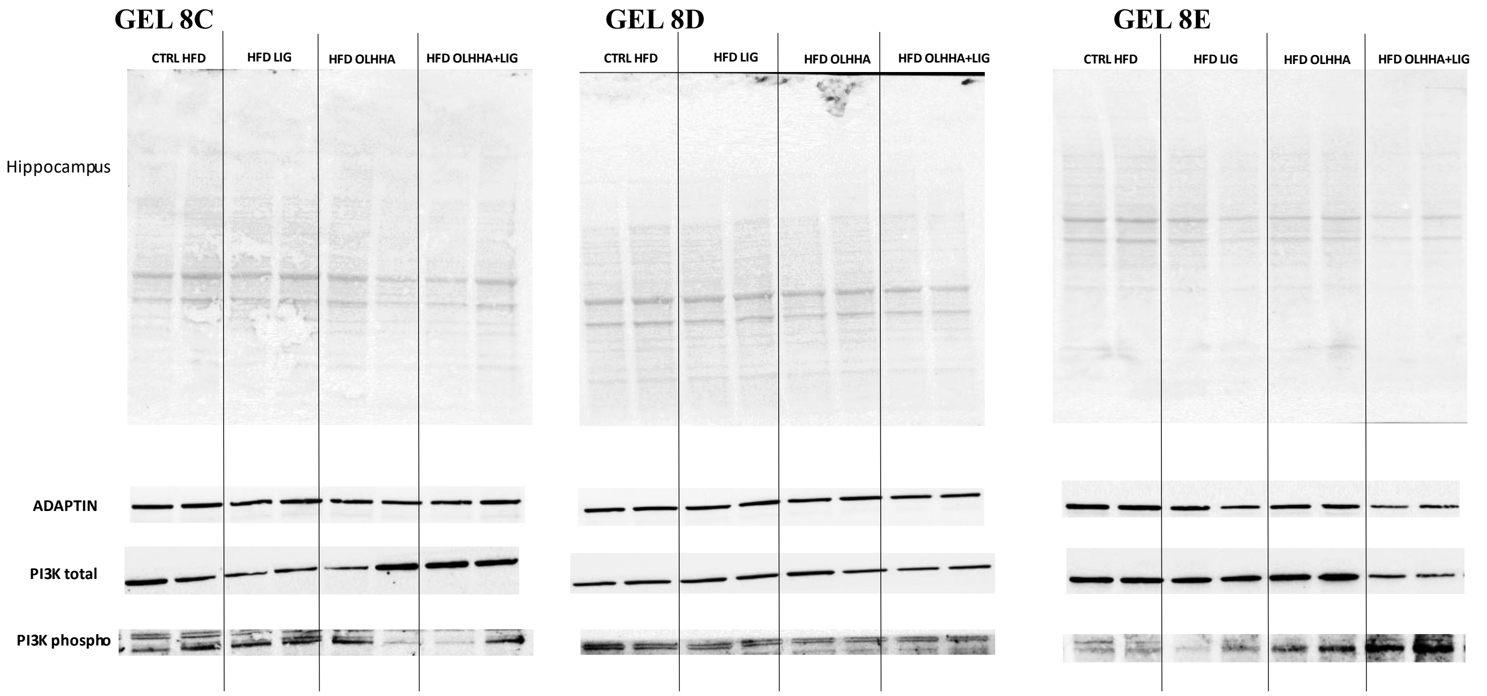

Supplementary figure S16: Western Blots of HIPPO, Ponceau and staining of membranes.

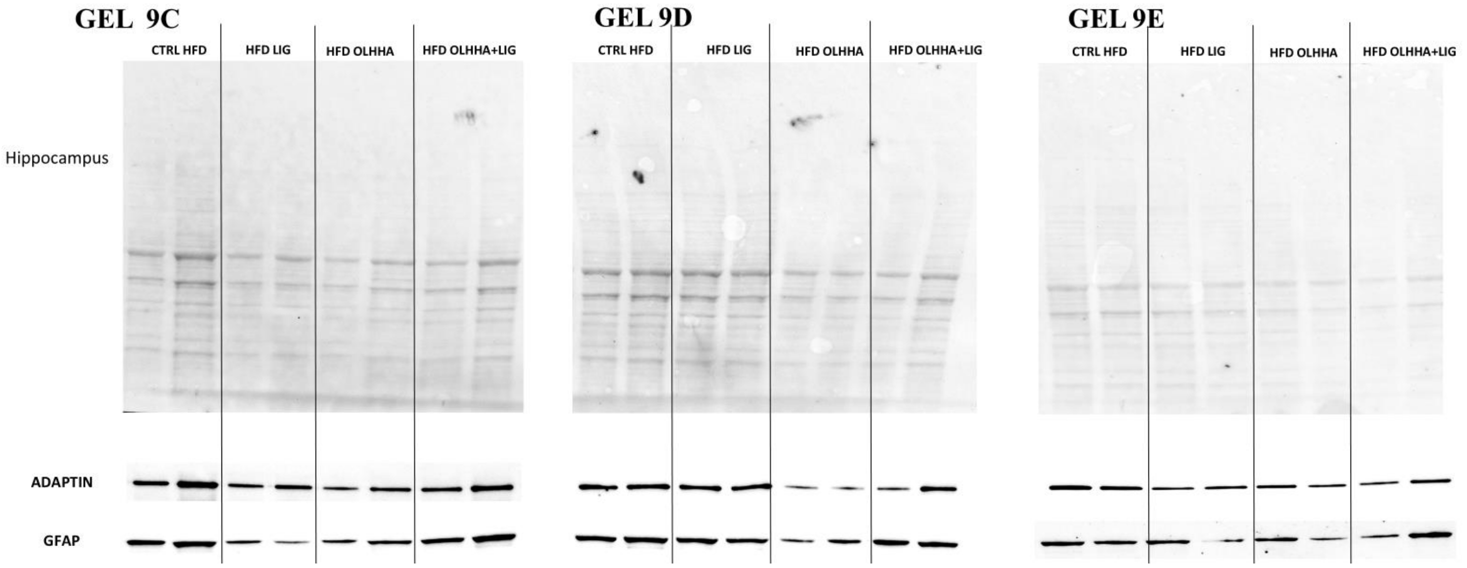

Supplementary figure S17: Western Blots of HIPPO, Ponceau and staining of membranes.

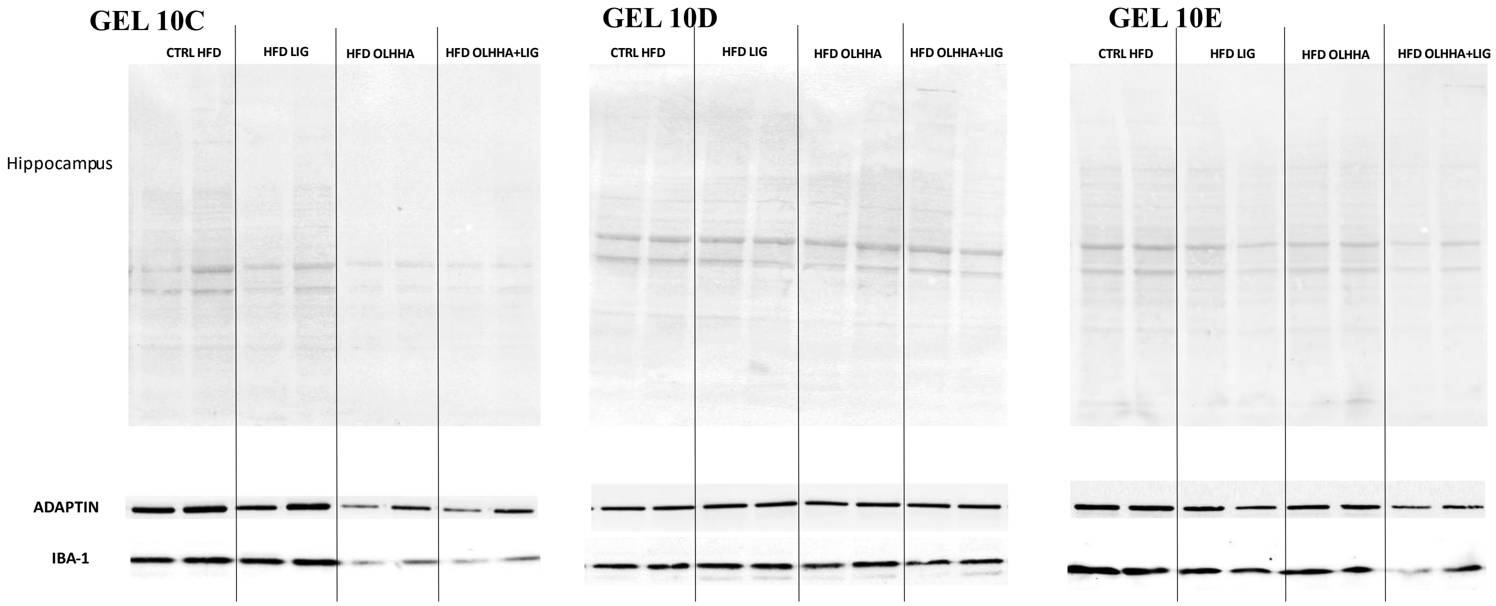

Supplementary figure S18: Western Blots of PFC, Ponceau and staining of membranes

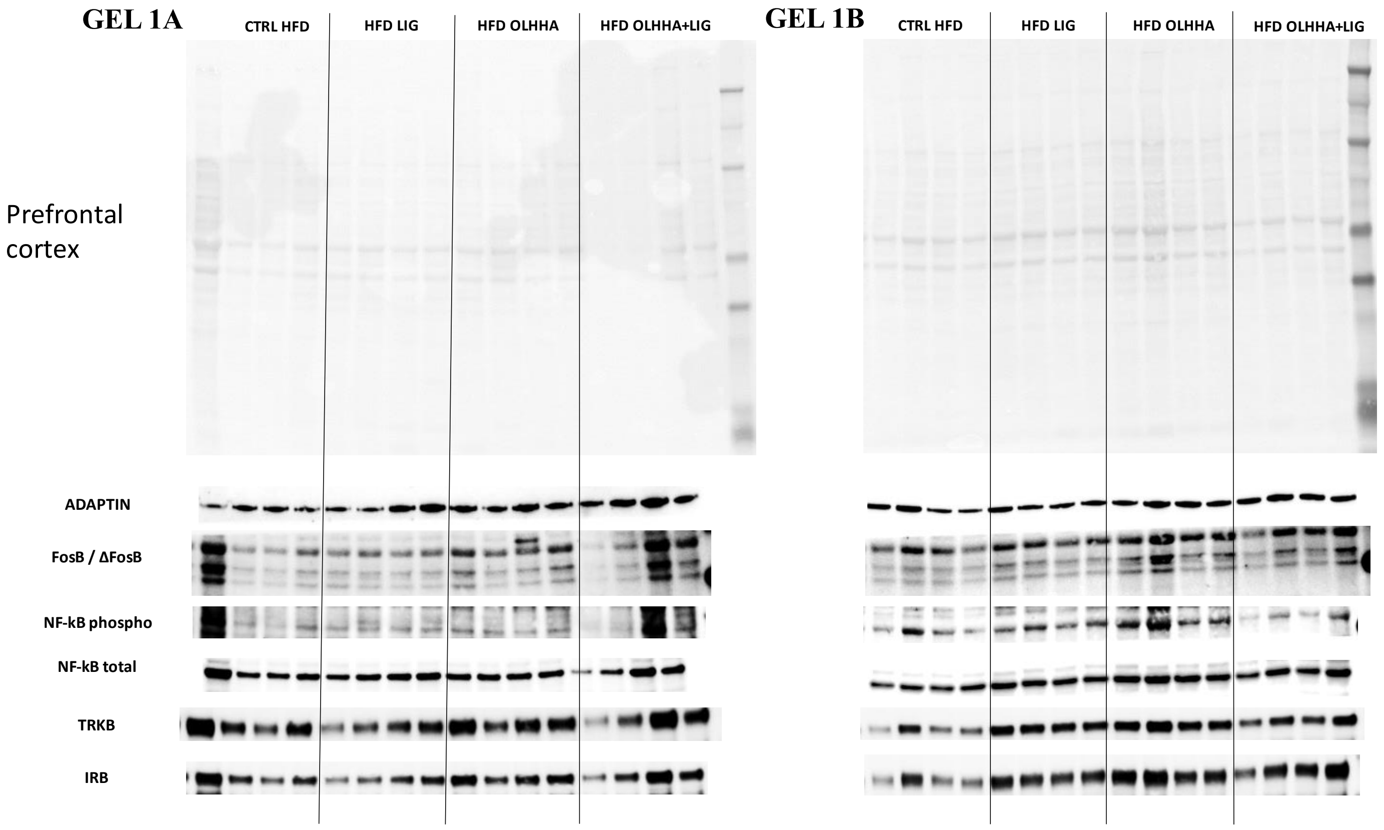

Supplementary figure S19: Western Blots of PFC, Ponceau and staining of membranes

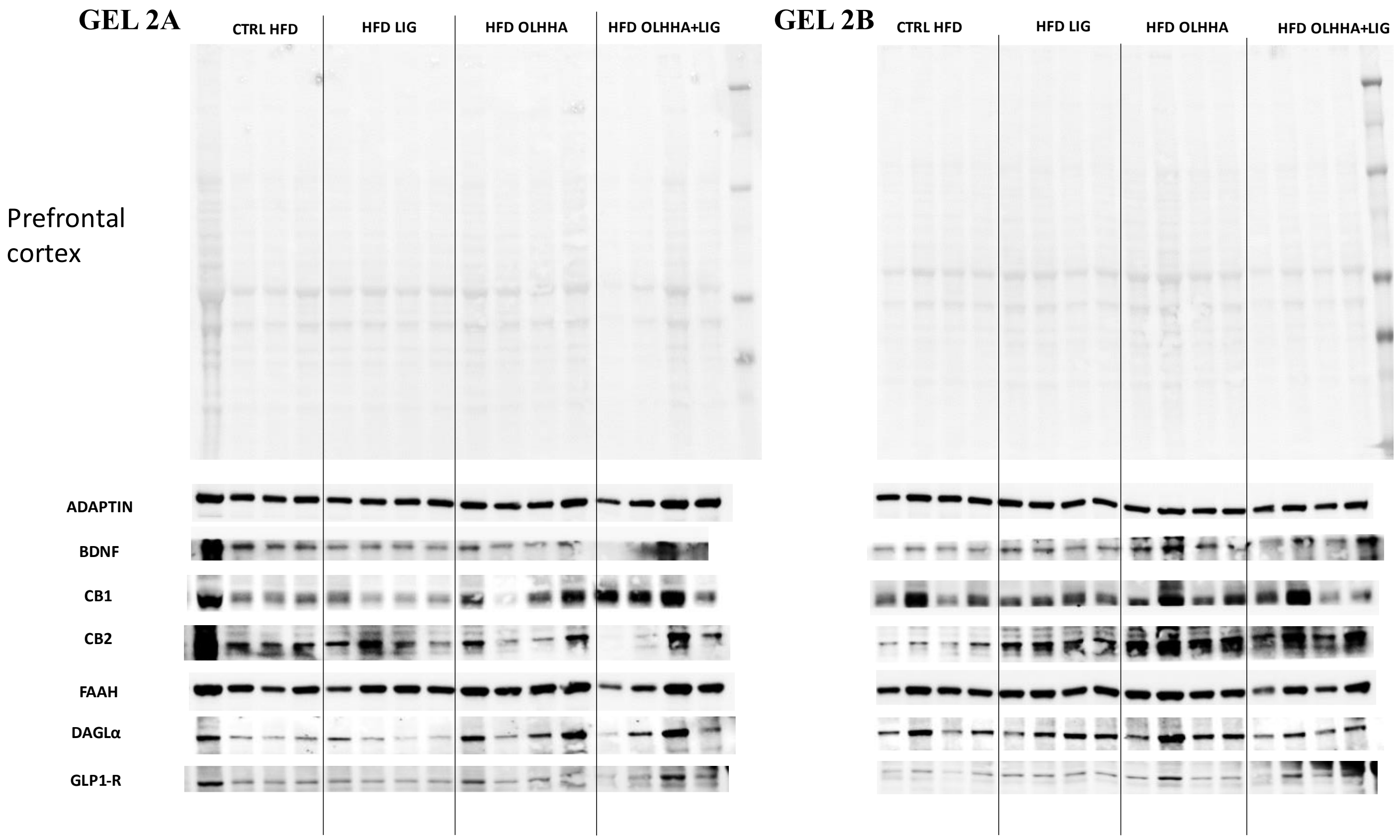

Supplementary figure S20: Western Blots of PFC, Ponceau and staining of membranes

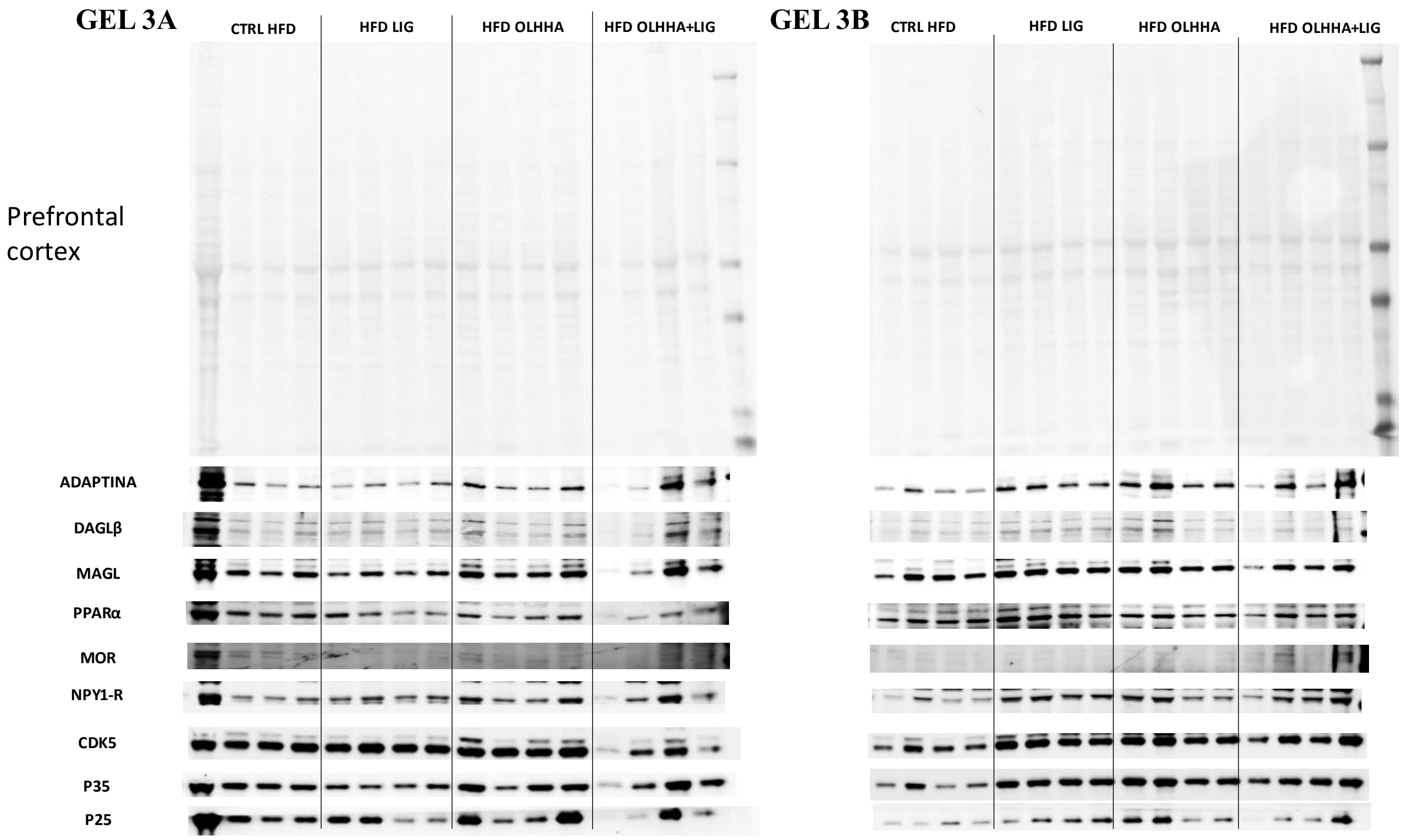

Supplementary figure S21: Western Blots of PFC, Ponceau and staining of membranes

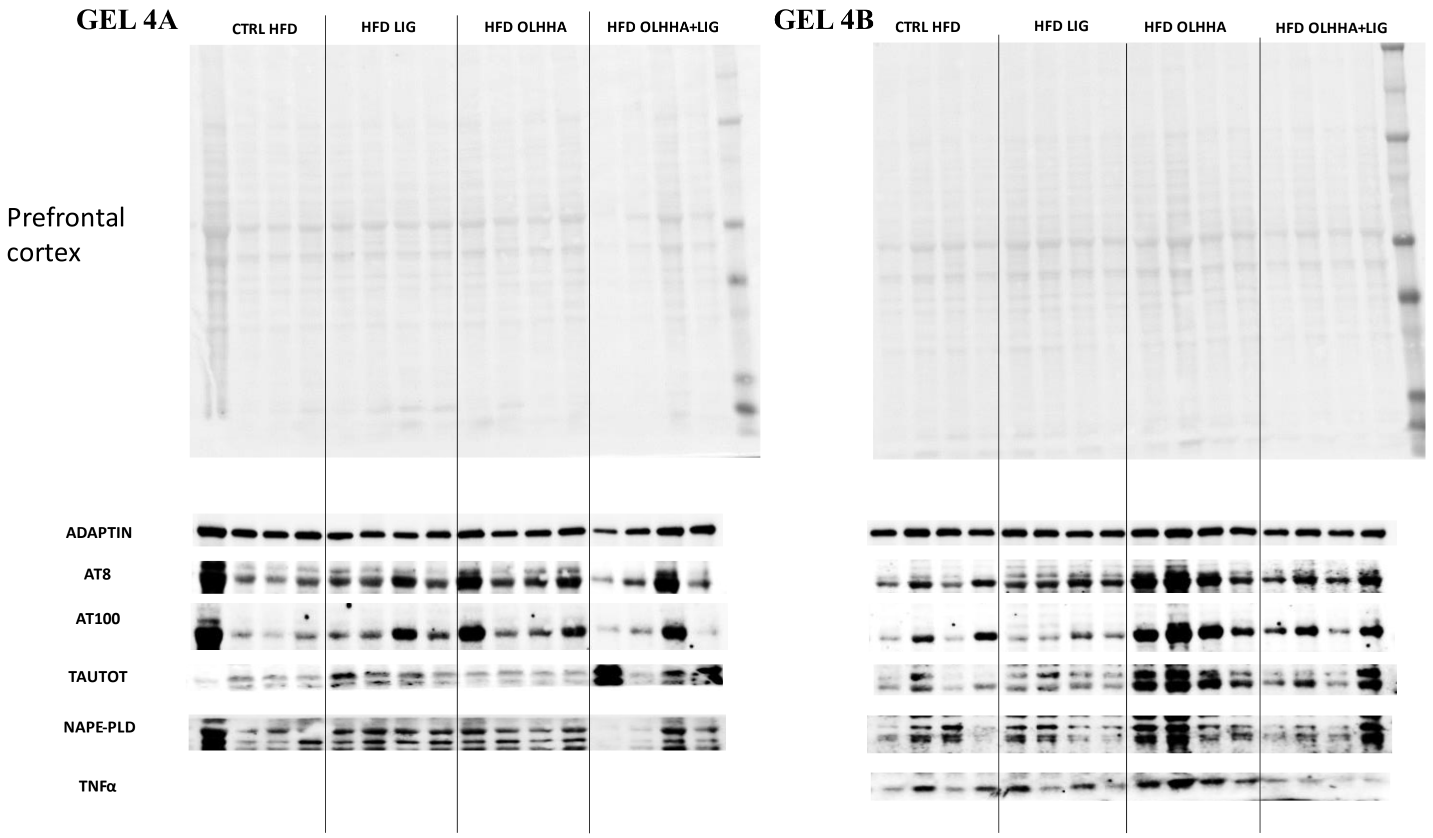

Supplementary figure S22: Western Blots of PFC, Ponceau and staining of membranes

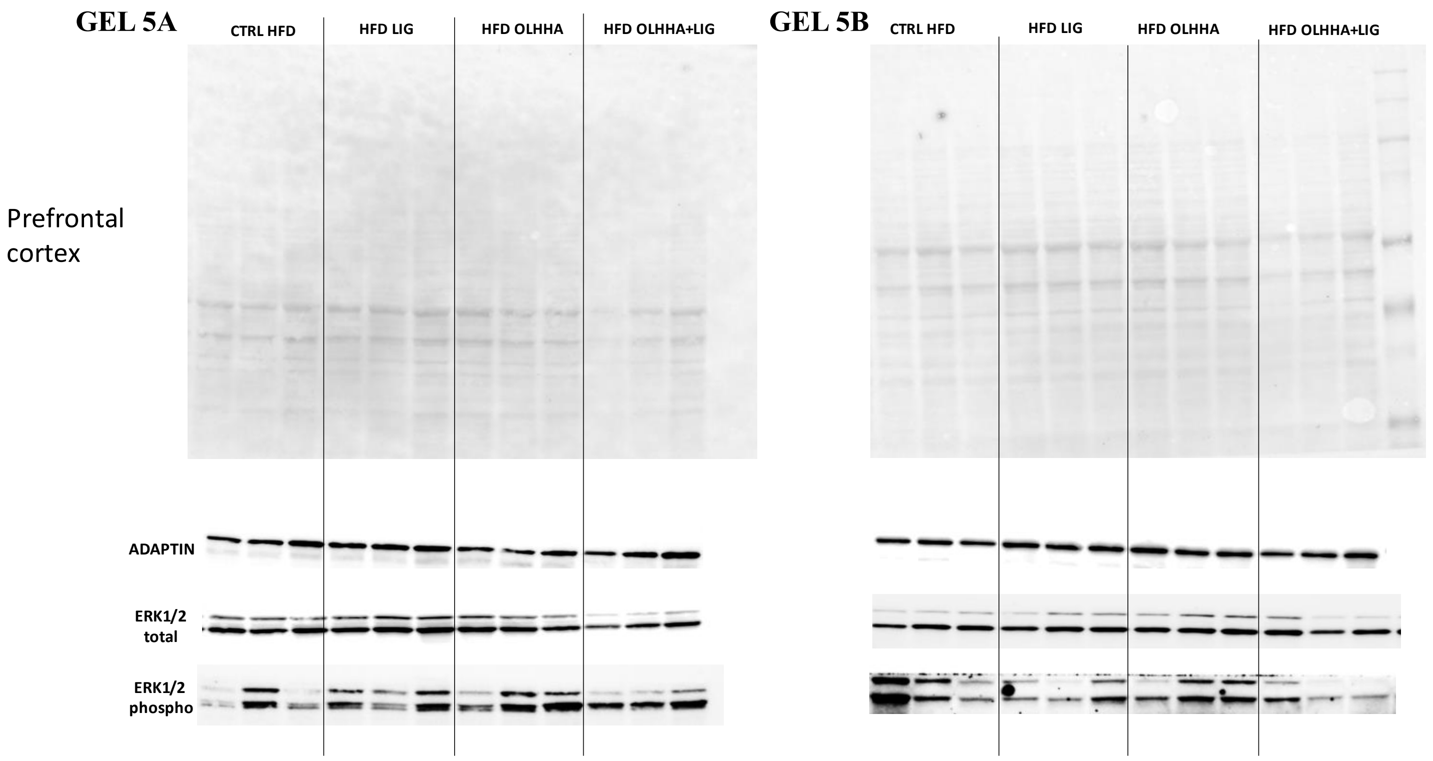

Supplementary figure S23: Western Blots of PFC, Ponceau and staining of membranes

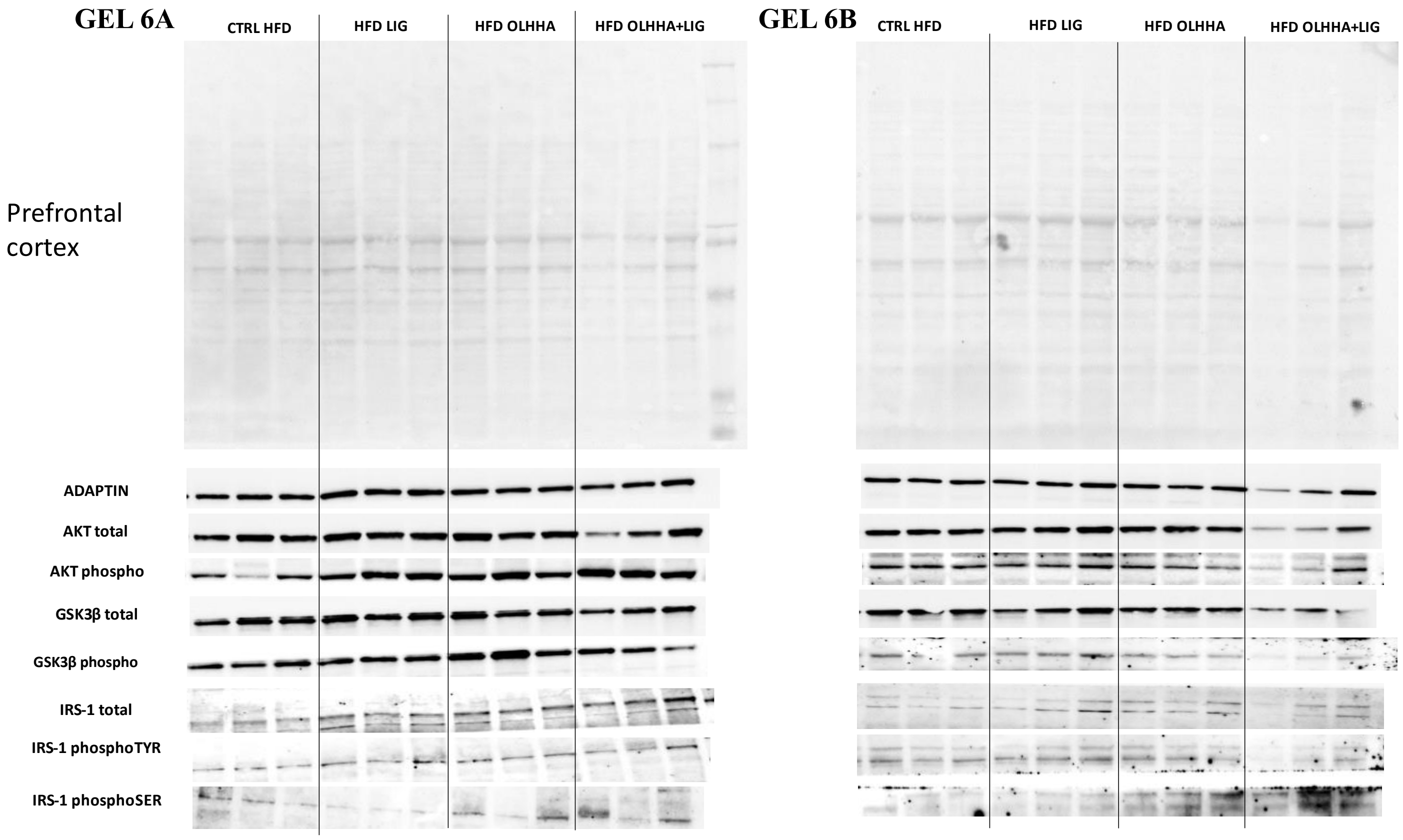

Supplementary figure S24: Western Blots of PFC, Ponceau and staining of membranes

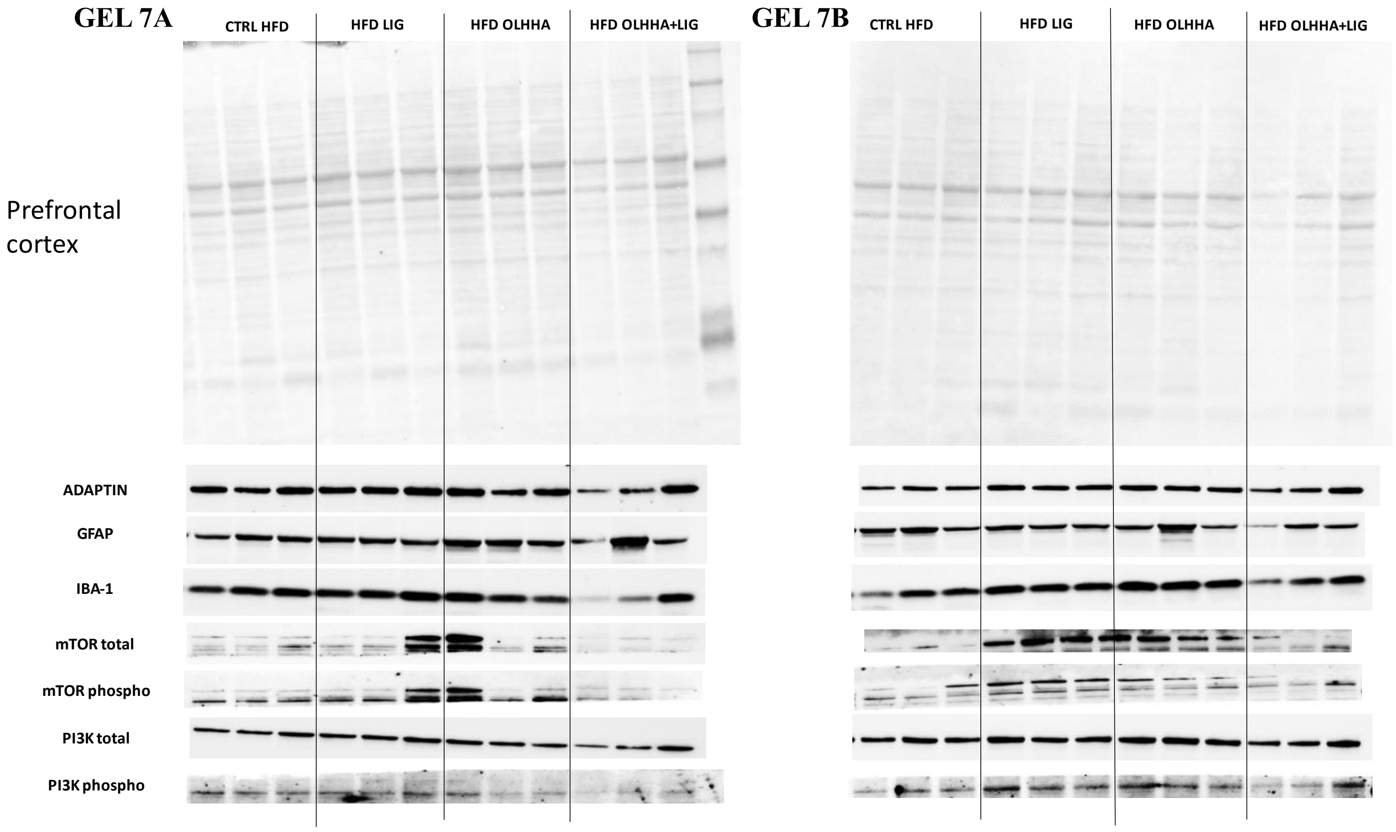

Supplementary figure S25: Complementary Western Blots

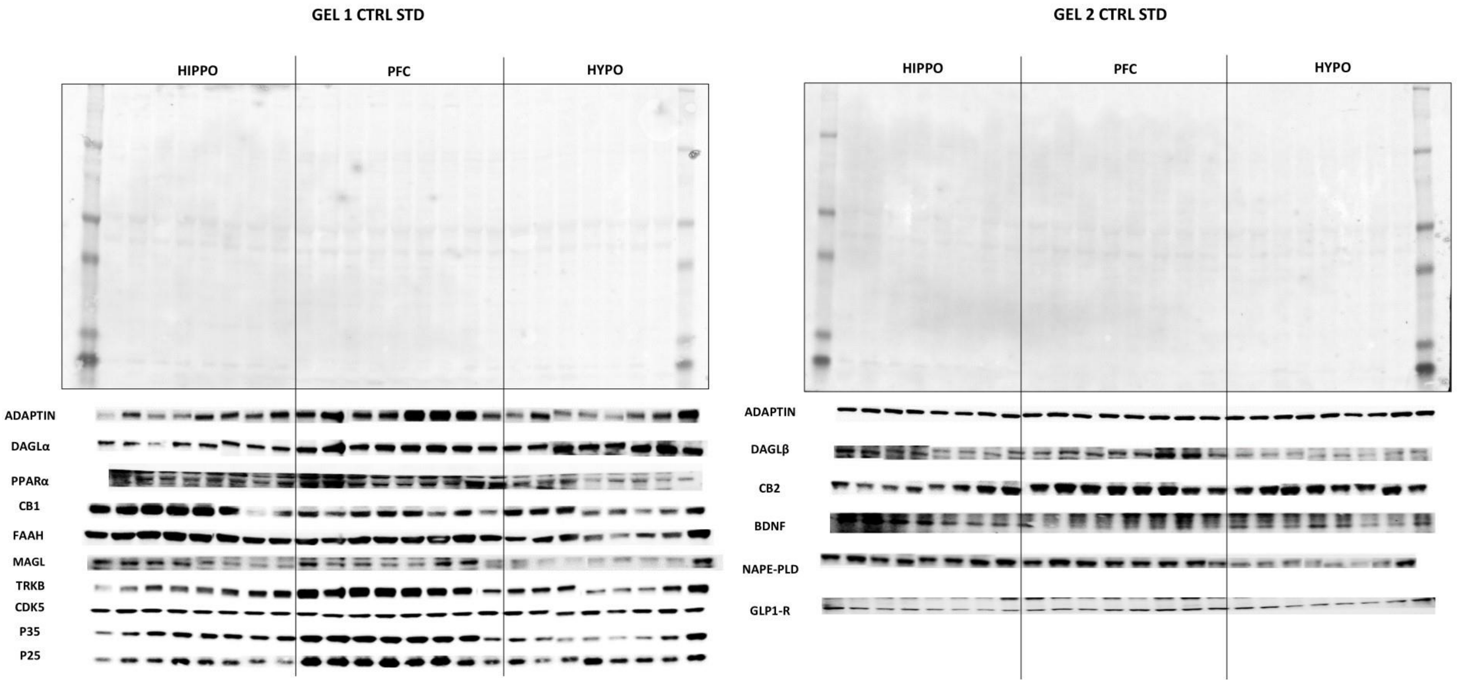

Supplementary figure S26: Complementary Western Blots

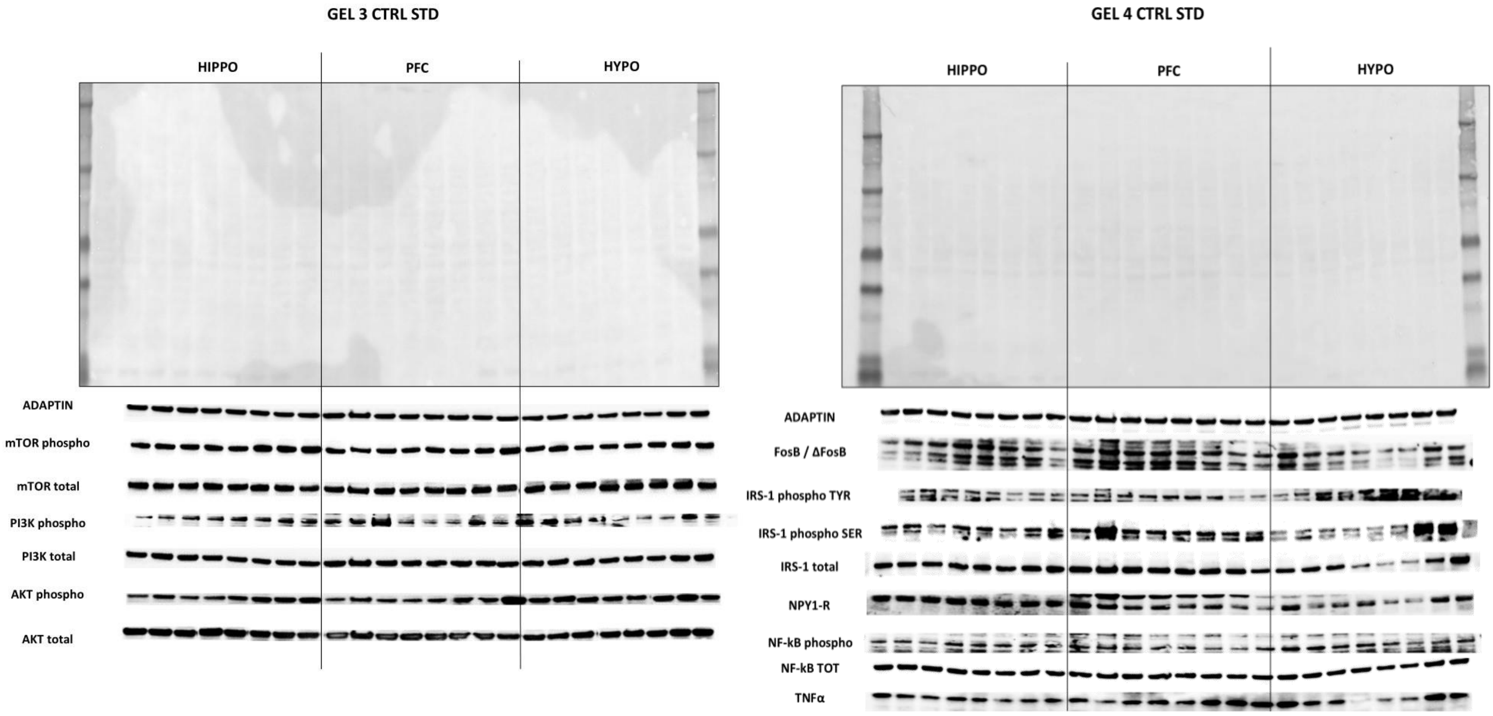

Supplementary figure S27: Complementary Western blots

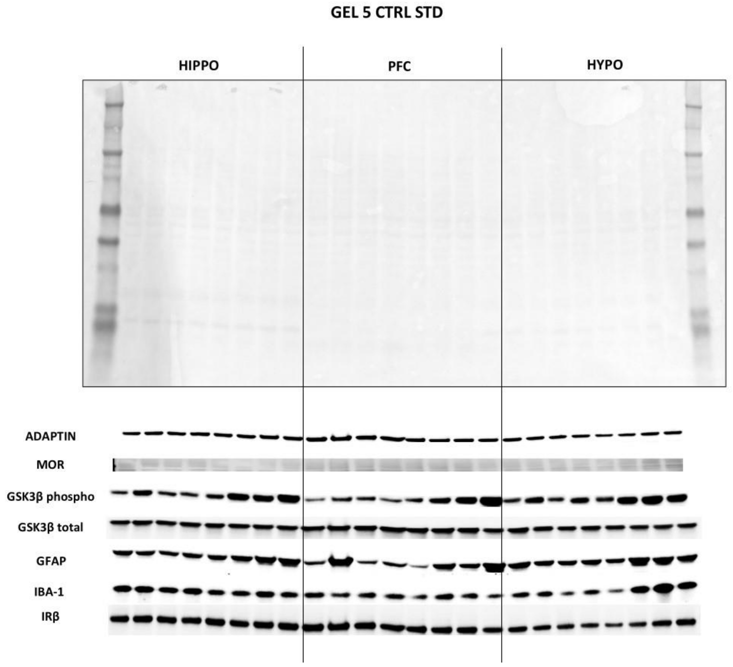

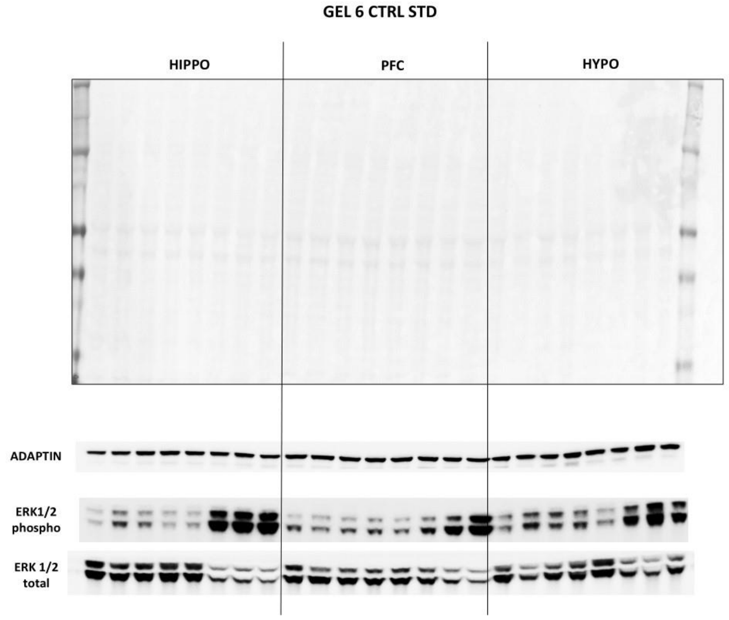

Supplementary figure S28: Complementary Western blots

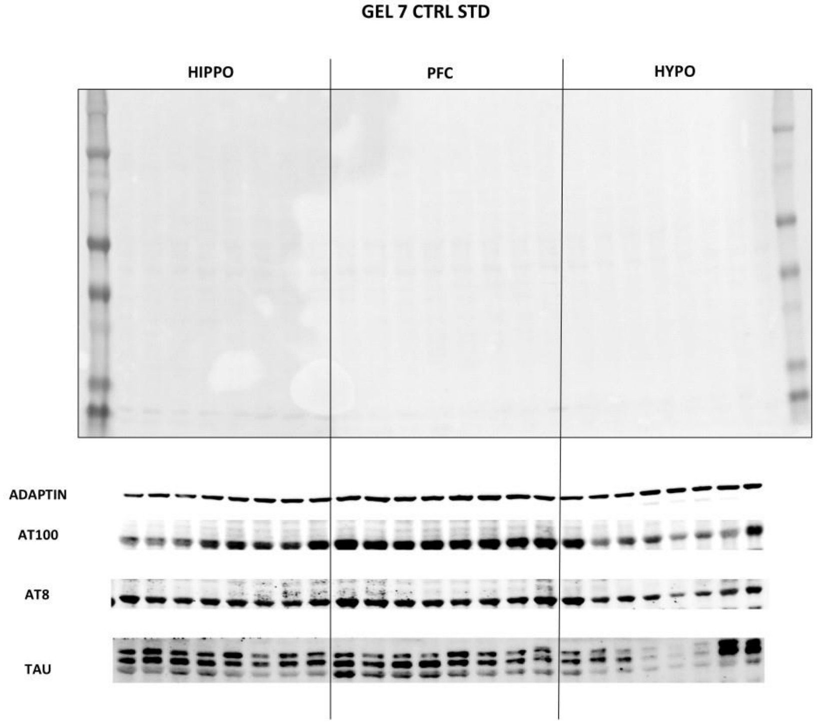

Table 5. List of primary antibodies used for protein expression by Western Blotting

Antibodies	*Manufacturing*	*Code*	*Dilution*	*Host*	*Molecular weight*
AKT	Cell signaling	9272	1/1000	Rabbit	60
AT100	Invitrogen	MN1060	1/500	Mouse	72-50
AT8	Invitrogen	203179	1/1000	Mouse	72-50
BDNF	Millipore Sigma	AB1534SP	1/500	Rabbit	28
CB1	Abcam	ab23703	1/200	Rabbit	60
CB2	Abcam	Ab3561	1/200	Rabbit	45
CDK5	Cell signaling	1234S	1/1000	Mouse	35
FosB	Santa Cruz	AGSC48	1/100	Rabbit	50-40
DAGLα	Biorbyt	Orb156533	1/100	Rabbit	110
DAGLβ	Biorbyt	Orb182976	1/100	Rabbit	72
ERK1/2	Cell signaling	4695S	1/1000	Rabbit	44
FAAH	Cayman	IT:101600	1/100	Rabbit	65
GLP1-R	Santa Cruz	390774	1/250	Mouse	53
GFAP	Thermofisher	MA5-12023	1/500	Mouse	50
GSK3β	Cell signaling	12456S	1/1000	Rabbit	51
IBA-1	Wako	019-19741	1/500	Rabbit	18
IRS-1 TOT	Millipore Sigma	06-248	1/1000	Rabbit	180
IRS-1 phospho SER	Cell signaling	SER612	1/1000	Rabbit	180
IRS-1 phospho TIR	Abcam	46800	1/1000	Rabbit	180
IRβ	Cell signaling	L55B10	1/1000	Mouse	80
MAGL	Abcam	Ab24701	1/500	Rabbit	36
mTOR	Cell signaling	2972S	1/1000	Rabbit	289
MOR	Abcam	ab-139054	1/200	Rabbit	75
NAPE-PLD	Abcam	Ab95397	1/1000	Rabbit	45
NF-kB	Cell signaling	8242S	1/1000	Rabbit	65
NPY1-R	Abcam	ab91262	1/1000	Rabbit	40
p35/25	Cell signaling	2680S	1/1000	Rabbit	35-25
phospho AKT	Cell signaling	9271	1/1000	Rabbit	60
phospho ERK1/2	Cell signaling	9101S	1/1000	Rabbit	44
phospho GSK-3β	Cell signaling	D85E12	1/1000	Rabbit	51
phospho mTOR	Cell signaling	2971S	1/1000	Rabbit	289
phospho NF-kB	Cell signaling	3033S	1/1000	Rabbit	65
phospho PI3K	Abcam	ab182651	1/1000	Rabbit	85
PI3K	Cell signaling	4257s	1/1000	Rabbit	85
PPARα	Abcam	ab15270	1/500	Rabbit	52
Tau total	Abcam	1020	1/1000	Mouse	72-50
TNF-α	Cell signaling	3707S	1/500	Rabbit	17-22
TrkB	Millipore Sigma	07-225	1/250	Rabbit	140
γ-adaptina	BD transduction	610385	1/2000	Mouse	100
